# *Enterococcus faecalis* Prophage Dynamics and Contributions to Pathogenic Traits

**DOI:** 10.1371/journal.pgen.1003539

**Published:** 2013-06-06

**Authors:** Renata C. Matos, Nicolas Lapaque, Lionel Rigottier-Gois, Laurent Debarbieux, Thierry Meylheuc, Bruno Gonzalez-Zorn, Francis Repoila, Maria de Fatima Lopes, Pascale Serror

**Affiliations:** 1 INRA, UMR1319 Micalis, Jouy-en-Josas, France; 2 AgroParisTech, UMR Micalis, Jouy-en-Josas, France; 3 ITQB, Universidade Nova de Lisboa, Oeiras, Portugal; 4 Institut Pasteur, Molecular Biology of the Gene in Extremophiles Unit, Department of Microbiology, Paris, France; 5 Dpto. de Sanidad Animal, Facultad de Veterinaria and VISAVET, Universidad Complutense de Madrid, Madrid, Spain; 6 IBET, Oeiras, Portugal; Uppsala University, Sweden

## Abstract

Polylysogeny is frequently considered to be the result of an adaptive evolutionary process in which prophages confer fitness and/or virulence factors, thus making them important for evolution of both bacterial populations and infectious diseases. The *Enterococcus faecalis* V583 isolate belongs to the high-risk clonal complex 2 that is particularly well adapted to the hospital environment. Its genome carries 7 prophage-like elements (V583-pp1 to -pp7), one of which is ubiquitous in the species. In this study, we investigated the activity of the V583 prophages and their contribution to *E. faecalis* biological traits. We systematically analyzed the ability of each prophage to excise from the bacterial chromosome, to replicate and to package its DNA. We also created a set of *E. faecalis* isogenic strains that lack from one to all six non-ubiquitous prophages by mimicking natural excision. Our work reveals that prophages of *E. faecalis* V583 excise from the bacterial chromosome in the presence of a fluoroquinolone, and are able to produce active phage progeny. Intricate interactions between V583 prophages were also unveiled: i) pp7, coined EfCIV583 for *E. faecalis* chromosomal island of V583, hijacks capsids from helper phage 1, leading to the formation of distinct virions, and ii) pp1, pp3 and pp5 inhibit excision of pp4 and pp6. The hijacking exerted by EfCIV583 on helper phage 1 capsids is the first example of molecular piracy in Gram positive bacteria other than staphylococci. Furthermore, prophages encoding platelet-binding-like proteins were found to be involved in adhesion to human platelets, considered as a first step towards the development of infective endocarditis. Our findings reveal not only a role of *E. faecalis* V583 prophages in pathogenicity, but also provide an explanation for the correlation between antibiotic usage and *E. faecalis* success as a nosocomial pathogen, as fluoriquinolone may provoke release of prophages and promote gene dissemination among isolates.

## Author Summary

*Enterococcus faecalis* is a member of the core-microbiome of the human gastrointestinal tract. In the last decades however, this bacterial species has emerged as a major cause of hospital-acquired infections worldwide. Some isolates are particularly adapted to the hospital environment, and this adaptation was recently linked with enrichment in mobile genetic elements including prophages, which are chromosomal integrated genomes of bacterial viruses. We characterized the biological prophage activity in an *E. faecalis* strain of clinical origin that harbors 7 prophages. Six active prophages exhibit intricate interactions, one of which is involved in a molecular piracy phenomenon. We also established, for the first time, a direct correlation between prophage and adhesion to human platelets, an initial step towards infective endocarditis. Finally, we showed that fluoroquinolone increases prophage activity and can thus contribute to horizontal gene spreading. Overall, we provide evidence that prophages are key players in *E. faecalis* evolution towards pathogenicity.

## Introduction

Acquisition of external DNA by horizontal gene transfer and gene loss are major driving-forces of bacterial genome evolution. Temperate bacteriophages contribute actively to such evolution as they integrate into and excise from the bacterial chromosome [1]. They also mediate horizontal gene transfer by transduction within and across bacterial species [2], [3]. By doing so, the integration of a temperate phage into the bacterial genome can provide new genetic properties to the bacterial host, and under some circumstances leads to the emergence of new pathogens within species, as shown for *Corynebacterium diphtheriae*, *Escherichia coli* and *Vibrio cholerae* [4]–[6].

Temperate phages contribute to bacterial fitness or virulence in at least three ways: introduction of fitness factors, gene disruption, and lysis-mediated competitiveness [7]. Import of fitness factors is also referred to as lysogenic conversion, which confers new traits to the targeted bacterium by providing genes that are not essential for the phage life cycle. Over the last few years, a plethora of prophage-associated genes that contribute to various aspects of bacterial pathogenesis has been identified [8], [9]. They encode functions such as ADP-ribosyltransferase toxins in *Pseudomonas aeruginosa* and *V. cholerae* [10], [11], detoxifying enzymes, e.g. *sod*C in *E. coli* O157 [12], type III effector proteins such as *sop*E, *sse*I, *ssp*H1 in *Salmonella enterica* [13]–[15], among many others (for a review, see [7]). In a different scenario, integration of a prophage may modify bacterial virulence or adaptability by disrupting bacterial genes or operons such as the *Staphylococcus aureus* beta-toxin-encoding gene inactivated upon integration of bacteriophage φ13 [16]. A third typical contribution of prophages to bacterial fitness is related to their capacity to excise from the chromosome in a fraction of the bacterial population. This excision, followed in most cases by induction of the phage lytic cycle can be beneficial for the surviving population. For example, excision of a *Listeria monocytogenes* prophage is sufficient to restore a functional transcriptional regulator promoting escape from the phagosome, and thus intracellular growth [17]. In other cases, a complete lytic cycle is required for the expression and release of Shiga toxins in *E. coli* [18] or for promoting adhesion to human platelets via PblA and PblB, two platelet-binding proteins that are integral part of the phage SM1 tail from *Streptococcus mitis* [19].

Prophage-like elements are not all functional in the sense that they fail to give progeny without the presence of helper elements. However, they can still harbor functional genes that contribute to their DNA mobilization and/or to bacterial host by providing active genes such as *S. aureus* toxins or *E. coli* effectors [20]–[22]. Such is the case of the Phage-Related Chromosomal Islands (PRCIs) of some Gram-positive bacteria that are mobile genetic elements, initially described as *S. aureus* pathogenicity islands (SaPIs). They encode mobilization functions as well as the toxic shock toxin, and other virulence and antibiotic resistance genes [20], [23]. They can also modulate host gene activity by dynamic excision and reintegration like the PRCI SpyCI of *Streptococcus pyogenes* [24]. PRCIs are mobilized by hijacking structural proteins of a helper phage to form specific-virions [25]. The genomic organization and the current knowledge of the molecular mechanisms of these pirate elements have been reviewed recently [26], [27]. Excision of SaPIs from the bacterial chromosome is induced upon infection by a helper phage or by induction of an endogenous prophage [28]. Following excision, SaPIs self replicate as concatemers, are packaged as monomers and multimers within small and large capsids, respectively, made of helper phage proteins [29], [30]. Redirection of helper phage proteins by SaPIs has been associated with interference mechanisms, which differ between SaPI elements [31], [32]. While PRCIs have been recently predicted in other gram-positive bacteria *in silico* [26], demonstration of their activity is still pending.

Beyond the understanding of the involvement of individual prophages in bacterial strain phenotypes and ecology, a few studies have started to tackle the more complex question of the impact of polylysogeny on bacterial physiology. For example cryptic prophages of *E. coli* improve growth, contribute to protection against antibiotics or stress and increase virulence or biofilm formation [33], [34]. On the other hand, temperate phages contribute to virulence of *S. enterica* [14] and *S. aureus* [35], and confer competitive fitness to *S. enterica* [36]. Polylysogeny often leads to intricate phenomenon of prophage interferences that are likely to influence behavior of the bacterial host as shown in *E. coli* and *S. enterica* [14], [33], [34].

*Enterococcus faecalis* is a low-GC Gram-positive bacterium whose primary habitat is the gastrointestinal tract of a wide range of animals and humans. This member of the core human microbiota [37] exhibits different lifestyles. It is commonly found in diverse environments including food, water, soil and plants, but it is also associated with life threatening infections. *E. faecalis* ranks among the leading causes of hospital acquired bacterial infections, and causes mostly urinary tract and intra-abdominal infections, infective endocarditis and bacteremia [38]. Epidemiological studies have revealed few enriched clonal complexes (CCs) of multi-drug resistant colonizing and/or invasive isolates among hospital-associated strains [39], [40]. Of these high-risk enterococcal clonal complexes, CC2 isolates are particularly well adapted to hospital environment and associated with invasive disease [41]. The strain V583 belongs to CC2 and was the first vancomycin resistant isolate found in the United States [42]. The chromosome of V583 harbors seven prophage-like elements (V583-pp1 to V583-pp7, named hereafter pp1 to pp7), one of which (pp2) is found in all *E. faecalis* isolates and is considered to be part of the core genome [43], [44]. Interestingly, CC2-isolates are enriched in prophage-genes, supporting the idea that these mobile genetic elements may contribute to increased survival of CC2 isolates in the host [45]. Noticeably, *E. faecalis* polylysogeny has been reported recently in a collection of clinical isolates, which carried up to 5 distinct inducible phages [46], indicating that polylysogeny is not specific to the V583 isolate. Even though several phage-encoded potential fitness factors have been pointed out [43], [46], [47], the contribution of these prophages to the lifestyle of *E. faecalis* and to its biological traits remains largely unknown.

The aim of this study was to establish whether the *E. faecalis* V583 prophages are biologically active and impact the host strain phenotype. Out of the seven prophages predicted, we show that six are inducible, and four form infectious virions through a sophisticated regulatory network, which revealed the first enterococcal phage-related chromosomal island. Moreover, we demonstrate the contribution of pp1, pp4 and pp6 to human platelet adhesion, suggesting a role of *E. faecalis* prophages in the development of nosocomial infective endocarditis.

## Results

### Prediction of phage functions coded by the V583 prophages

**Figure 1:**
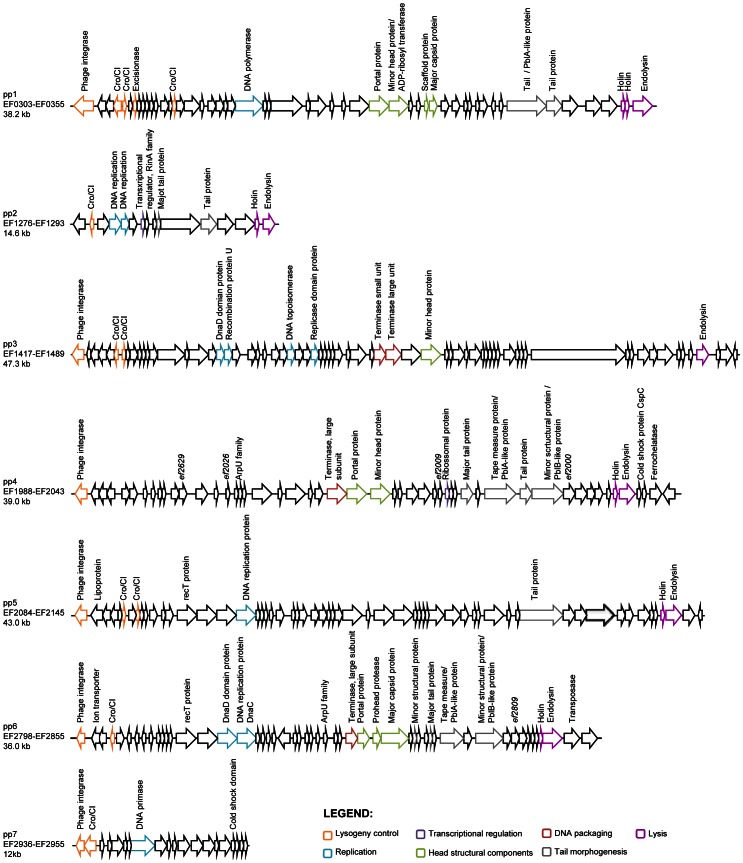
Genomic organization of *E. faecalis* V583 prophages. Open-reading frames are indicated by arrows. Only genes encoding predicted function are annotated. Colors correspond to the seven functional modules of temperate phages as depicted at the bottom right corner.

Generally, temperate phage genomes are organized in modules of genes corresponding to important functions for their life cycle, which facilitates temporal order of gene transcription. Six modules are classically recognized: lysogeny, replication, transcriptional regulation, head and tail morphogenesis, DNA packaging and lysis [48], [49]. The chromosome of *E. faecalis* V583 harbors seven prophage-like elements [43]. Table 1 summarizes the presence and absence of functions relevant to the identifiable modules on V583 prophages. Five of the seven prophages (pp1, pp3, pp4, pp5 and pp6) contain genes for all modules suggestive of genome completeness. All five contain an integrase, but only pp1 bears a recognizable excisionase function, which enables prophages to excise from the bacterial chromosome. However, it cannot be excluded that integrases use alternative accessory proteins such as recombination directionality factors allowing them to mediate prophage integration and excision [50]. Thus, prophages 1, 3, 4, 5 and 6 seem to have all necessary functions to undertake a complete lytic cycle (Figure 1). Among them, pp3 and pp5 have similar gene organization. Genes encoding potential fitness factors, namely homologs of *S. mitis* platelet binding proteins PblA and PblB (pp1, pp4 and pp6), a ferrochelatase (pp4) and a more recently identified toxin ADP-ribosyltransferase (pp1) have been predicted [43], [47]. The genomes of pp2 and pp7 are particularly small in comparison with the five other prophages (∼12 Kb versus >36 Kb). According to recent reports, pp2 belongs to the *E. faecalis* core genome [44], [45], [51], and the lack of an integrase gene suggests that pp2 is a remnant phage. Prophage 7 encodes an integrase and a replication related protein, however it lacks the head and tail morphogenesis modules essential for capsid formation as well as genes involved in DNA packaging and lysis. These predictions suggest that pp7 is either defective or belongs to the family of the phage-related chromosomal islands (PRCIs) predicted in Gram-positive bacteria, including *E. faecalis* [26]. Altogether, five of the V583 prophages are predicted to form active particles autonomously.

**Table 1:** Summary of predicted essential phage functions in V583 prophages.

V583 prophage	Prophage size (kb)	Prophage localization	Lysogeny			Replication	Morphogenesis			DNA packaging	Lysis
			Repressor (CI)/anti-repressor (Cro)	Integrase	Excisionase		Portal	Head	Tail		
pp1	38.2	*ef0303-ef0355*	**+**	**+**	**+**	**+**	**+**	**+**	**+**	**+**	**+**
pp2	14.6	*ef1276-ef1293*	**+**	**−**	**−**	**+**	**−**	**−**	**+**	**−**	**+**
pp3	47.3	*ef1417-ef1489*	**+**	**+**	**−**	**+**	**−**	**+**	**+**	**+**	**+**
pp4	39.0	*ef1988-ef2043*	**+**	**+**	**−**	**+**	**+**	**+**	**+**	**+**	**+**
pp5	43.0	*ef2084-ef2145*	**+**	**+**	**−**	**+**	**−**	**+**	**+**	**+**	**+**
pp6	36.0	*ef2798-ef2855*	**+**	**+**	**−**	**+**	**+**	**+**	**+**	**+**	**+**
pp7^a^	12.0	*ef2936-ef2955*	**+**	**+**	**−**	**+**	**−**	**−**	**−**	**−**	**−**

a Renamed EfCIV583 in this work.

### Prophages 1, 3, 4, 5 and 7 excise from the chromosome

Prophages excise from the bacterial chromosome by inactivation of their repressor triggered either spontaneously or by a signal, which frequently depends on the induction of the SOS response [52]. To confirm our predictions on prophage activity, we tested the ability of the V583 prophages under various environmental stresses to accomplish the four major steps of temperate phage life cycle: excision, replication, DNA packaging and production of infectious particles.

**Figure 2:**
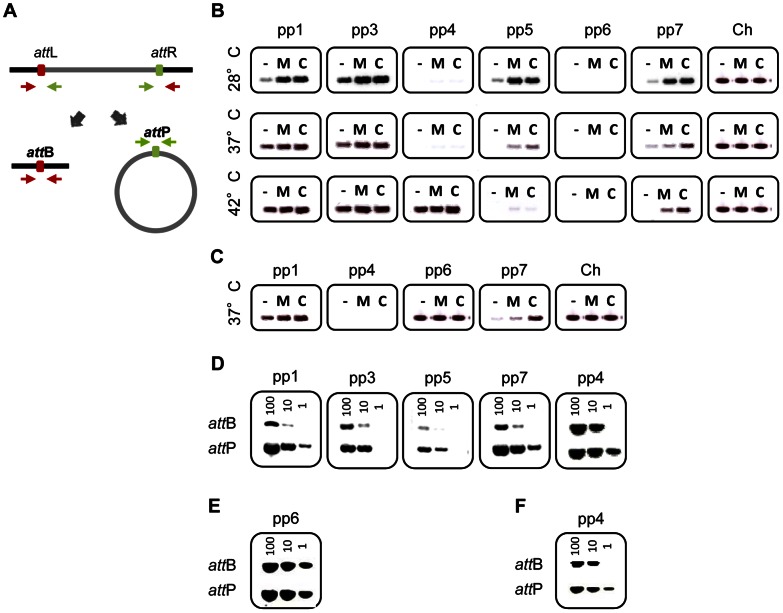
Prophage excision and replication. Agarose gel analysis of prophage excision and circularization products corresponding to *attB* and *attP* regions, respectively, probed by PCR. (A) Experimental approach: two sets of primers were used to detect prophage excision from the chromosome. The first set in red targets the excision site on the chromosome (*attB*) and the second set in green targets prophage circular forms (*attP*). (B) Prophage excision products corresponding to *attB* region were probed by PCR in WT cultures induced with 2 µg/ml of mitomycin C (M), or ciprofloxacin (C) or uninduced (−) at 28, 37 and 42°C. Ch corresponds to amplification of a strain specific chromosomal gene. (C) Prophage excision products in strain *pp3^−^ pp5^−^* at 37°C. (D–F) Excision and circularization products probed by semi-quantitative PCR on 100, 10 and 1 pg of total bacterial DNA prepared from cultures of WT (D) and strains *pp3^−^ pp5^−^* (E) *pp4^+^* (F) induced for 2 h with 2 µg/ml of ciprofloxacin at 37°C except for detection of pp4 products in the WT strain that were obtained from DNA prepared after induction at 42°C. Twenty and 32 PCR cycles were used to amplify products of pp1 and pp7 and products of pp3, pp4 and pp5, respectively. These results are representative of three independent experiments.

We studied the activity of V583 prophages in the strain VE14089, which is a V583 derivative cured of its plasmids, and referred to as WT hereafter, a genetically tractable strain compared to the original V583 [53]. To determine whether prophages were able to excise from the chromosome, bacteria were challenged either with chemical compounds known to trigger prophage induction through SOS response and/or formation of reactive oxygen species (mitomycin C, ciprofloxacin, thrimetoprim, ampicillin), or with varying temperatures (28, 37 and 42°C) for 2 hours. Total DNA was recovered and analyzed by PCR to search for expected products of chromosomal excision and prophage circularization, referred as *attB* and *attP* region, respectively (Figure 2A). Results of amplification of the *attB* region resulting from prophage excision obtained with or without mitomycin C and ciprofloxacin are presented in Figure 2B. The excision of pp1, pp3, pp5 and pp7 was already detected under non-inducing conditions, indicating a basal natural excision in laboratory growth conditions. Yet, prophages responded differently to environmental challenges. While pp3 was equally induced at 28, 37 and 42°C, natural induction of pp1 increased with temperature but those of pp5 and pp7 decreased with temperature. Strikingly, excision of pp4 was detected only at high temperature (42°C) (Figure 2B). Both mitomycin C and ciprofloxacin increased or triggered pp1, pp3, pp4, pp5 and pp7 excision from the bacterial chromosome at all tested temperatures. Trimethoprim challenge induced prophages similarly to ciprofloxacin, while ampicillin had no effect on prophages induction (data not shown). No excision of pp2 and pp6 was detected in any of the tested conditions. The PCR amplification fragments of the excision and integration sites were sequenced and confirmed the *in silico* predictions from V583 genomes of the attachment (*att*) core sequences (Table 2). This information was further used to reconstitute the prophage integration sites upon deletion by homologous recombination (see below). Note that excision of pp4 restores an open reading frame in an operon encoding competence-like genes [54], and that pp7 integrates in the promoter region of a putative xanthine/uracil permease gene. Together, these results revealed that pp1, pp3, pp4, pp5 and pp7 excise from the chromosome, and that all prophages, but pp3 show different responses to environmental cues such as temperature and antibiotics, suggesting potential population heterogeneity of WT strain depending on each growth condition. However, in all conditions tested pp2 and more surprisingly pp6, were not excised.

**Table 2:** Prophage *att* core sequence predicted and confirmed experimentally from V583 genome^a^.

Prophage	Genes	Integration site	Sequence 5′-3′
pp1	*ef0303-ef0355*	3′ end of *ef0302*	CCTTGGGATCCAATGGG
pp3	*ef1417-ef1489*	3′ end of *ef1416*	ACAAACGCAACATGTTCGCTTTATTAGGTAAACCAGG
pp4	*ef1988-ef2043*	Within *cglD*-like^b^ gene	CCACTCCCCATCTGAAATT
pp5	*ef2084-ef2145*	3′ end of *tRNA-Thr2*	GGCAGGTGGCT
pp6	*ef2798-ef2855*	Downstream of 3′ end of *ef2856*	TAAATTATTTAGTTTCACGGTGTAA
pp7^c^	*ef2936-ef2955*	Upstream of 5′ end of *ef2935*	TATTAATGAAACAACGTG

a Genome accession number: AE016830.

b *cglD* stands for for comG-like [54].

c Renamed EfCIV583 in this work.

### Prophages 3 and 5 inhibit excision of prophage 6

Because complex mechanisms of interference between prophages have been reported [31], [55], we wondered whether elimination of some prophages would facilitate excision of pp2 and pp6. For this purpose, we deleted successively prophages pp3 and then pp5 (see Materials and Methods) and then checked for pp2 and pp6 excision. Interestingly, pp6 was excised in strain *pp3* ^−^ *pp5* ^−^ deleted for pp3 and pp5 (Figure 2C), whereas it was not excised in strains deleted either for pp3 or pp5 alone (data not shown). While pp6 basal level of excision was not increased upon temperature or chemical challenge, this finding allowed us to determine the pp6 *att* core sequence (Table 2). Finally, prophage deletions were performed to generate strain *pp* ^−^ deleted for all prophages but pp2. Again, no excision of pp2 was detected validating that pp2 is a phage remnant. We conclude that pp6 excision is repressed by both pp3 and pp5. Thus we demonstrated that prophages carried by the V583 *E. faecalis* chromosome, with the exception of pp2, can excise and thereby may form phage progeny.

### Prophages 1, 3, 5 and 7 form infectious virions

**Figure 3:**
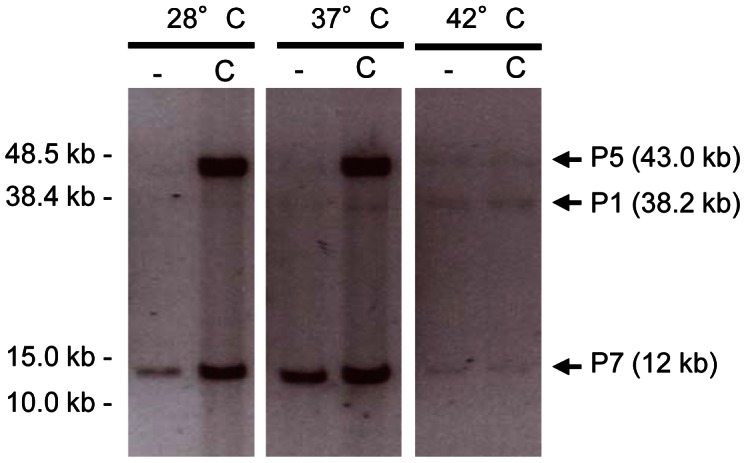
Detection of prophage packaged DNA. Encapsidated prophage DNA recovered from supernatant of WT cultures obtained at different temperatures 2 h after ciprofloxacin treatment at 2 µg/ml and detected by southern-blot hybridization with prophage specific probes. Non-treated and ciprofloxacin treated cultures correspond to lanes (−) and (C), respectively. DNA of P1, P5 and P7 is encapsidated at all temperatures under inducing conditions.

We then established which V583 prophages were able to replicate their genome after excision. Levels of both prophage circular forms and chromosomal excision regions from uninduced and ciprofloxacin-induced cultures were compared using semi-quantitative PCR. Circular forms of pp4 at 42°C, and pp1, pp3, pp5 and pp7 at 37°C were at least 10-fold more abundant than the corresponding chromosomal excision regions, respectively (Figure 2D). In contrast, no replication activity was detected for pp6 in a *pp3* ^−^ *pp5* ^−^ strain (Figure 2E). To further investigate whether DNA of prophages could be packaged into phage particles, we precipitated phage particles from ciprofloxacin-induced cultures of wild-type strain at 28, 37 and 42°C and strain *pp3* ^−^ *pp5* ^−^ at 37°C and extracted packaged DNA. Samples of phage DNA were analyzed by FIGE followed by Southern-blot hybridization with prophage-specific probes. In all the tested conditions, packaged DNA of pp1 (38.2 kb), pp5 (43.0 kb) and pp7 (12 kb) were observed, showing that pp1, pp5 and pp7 DNAs were encapsidated whereas DNA of pp3, pp4, and pp6 was not detected (Figure 3). As pp7 does not encode its own capsid proteins (Table 1), it might need a helper phage to form particles like PRCIs (see below). Packaged DNA of pp5 and pp7 was less abundant at 42°C, as expected (see Figure 2B). While the absence of pp6 DNA-containing particles correlates with the lack of pp6 replication, non detection of particles of pp3 and pp4 DNA could be explained either because their DNA was not packaged or the techniques used were not appropriate to isolate the cognate phage particles.

Finally, to determine which *E. faecalis* prophages have kept full viral activity, we examined their ability to form infectious virions. As a way to recognize the different virions generated by the WT strain, we constructed a set of isogenic strains deleted for individual prophages, namely strains *pp1* ^−^, *pp3* ^−^, *pp4* ^−^, *pp5* ^−^, *pp6* ^−^ and *pp7* ^−^, which harbored the natural *att*B integration site of the deleted prophage previously determined (see Material and Methods) (Table S1). In a naïve scheme, such strains should be immune to superinfection by all phages except the one that no longer stands in the bacterial genome. Phage-deleted strains were infected with supernatants of ciprofloxacin-induced cultures from WT and *pp3* ^−^ *pp5^−^* strains (Table S3). Plaques were detected on strains *pp3* ^−^, *pp5* ^−^, and *pp7* ^−^, suggesting that particles containing pp3, pp5 or pp7 DNA are infectious. Interestingly, despite encapsidation of pp1 DNA (Figure 3), lytic activity of pp1 DNA-containing particles was not detected on strain *pp1* ^−^. This result suggested that either pp1 DNA-containing particles were non-infectious, or that strain *pp1* ^−^ was still immune to P1 (see below). No plaque formation was observed on indicator strains *pp4* ^−^ and *pp6* ^−^, indicating that although pp4 and pp6 were excised and pp4 replicated, these prophages are deficient for the formation of infectious particles.

Since phage interactions or interference could occur during particle or plaque formation we constructed monolysogen strains for each prophage, named *pp1* ^+^ to *pp7* ^+^, and tested the ability of their ciprofloxacin-induced supernatants to form infectious particles on a *pp* ^−^ strain deleted for all prophages (Table S3). The results confirmed that pp3 and pp5 produced infective virions, and that pp4 and pp6 did not. As expected, we confirmed that pp6 circular forms were detected in strain *pp6^+^* in uninduced conditions (data not shown). Prophage 4, which excision depends on a high temperature (42°C) in strain WT, excises readily and replicates at 37°C in strain *pp4^+^*, deleted of all prophages but pp4 (Figure 2F). This observation suggests that some of the other V583 prophages could interfere with pp4 excision at 37°C in the WT strain. Interestingly, supernatant of the pp1 monolysogen strain (*pp1* ^+^) formed plaques on strain *pp* ^−^, indicating that pp1 DNA containing particles were infectious in the absence of other prophages. In contrast, the pp7 monolysogen strain (*pp7* ^+^) failed to produce infectious particles, further supporting that pp7 requires a helper phage.

Despite the absence of visible lysis upon prophage-inducing treatments, we evaluated the effect of prophage induction on bacterial population by assessing the growth of the strains WT, *pp^−^*, *pp1^+^* and *pp3^+^ pp5^+^* 6 h after ciprofloxacin-mediated induction. Ciprofloxacin treatment of wild-type strain lowered the growth of approximately 10% compared to the untreated culture while similar treatment had no effect on strain *pp^−^* deleted for all prophages (Figure S1). Moreover, the strains *pp1^+^* and *pp3^+^ pp5^+^* showed significantly decreased biomass when treated with ciprofloxacin. These observations suggest that V583 prophages are induced or perform full lytic cycle in a fraction of the bacterial population only, thereby leading to a mixed population with different combination of excised prophages.

In sum, these results demonstrate that pp1, pp3, pp5 and pp7 produce infective virions in specific conditions. Despite their excision, pp4 and pp6 are unable to produce infectious particles. Noticeably, pp4 and pp6 genomes contain several pseudogenes located in the morphogenesis module (*ef2000*, *ef2009*, *ef2026*, *ef2029* for pp4 and *ef2809* for pp6) that could explain that these phages are defective in capsid assembly. While phages 1 (P1), 3 (P3) and 5 (P5) are autonomous, pp7 requires a helper phage to form infectious particles. P3 and P5 provide self-immunity to their bacterial host, and P1 shows cross-immunity with at least one of the other prophages.

### Prophage 7 requires P1 as a helper phage for encapsidation

Subordination of pp7 to a helper phage to form infectious particles was correlated with comparative genomic hybridization data (Akary and Serror, *unp. data*) and sequence analysis of available genomes, which indicate that pp7 is present only in few CC2 isolates that also carry pp1 whereas pp1 is sometimes present alone [45], [56]. Thus, we hypothesized that P1 acts as a helper phage of pp7. To test our hypothesis, we constructed strain *pp1* ^+^ *pp7* ^+^, which contains pp1 and pp7 only (Table S1). Supernatants of ciprofloxacin-treated isogenic strains *pp1* ^−^ and *pp1* ^+^ *pp7* ^+^ were tested for plaque formation on the indicator strain *pp7* ^−^. While the dilysogen strain *pp1* ^+^ *pp7* ^+^ gave plaques, deletion of pp1 abrogated plaque formation, demonstrating that pp1 is necessary and sufficient for production of P7 virions. We conclude that the presently described pp7 corresponds to the phage-related *E. faecalis* chromosomal island, predicted by Novick and collaborators [26], and rename pp7 as EfCIV583 for *E. faecalis* chromosomal island V583.

**Figure 4:**
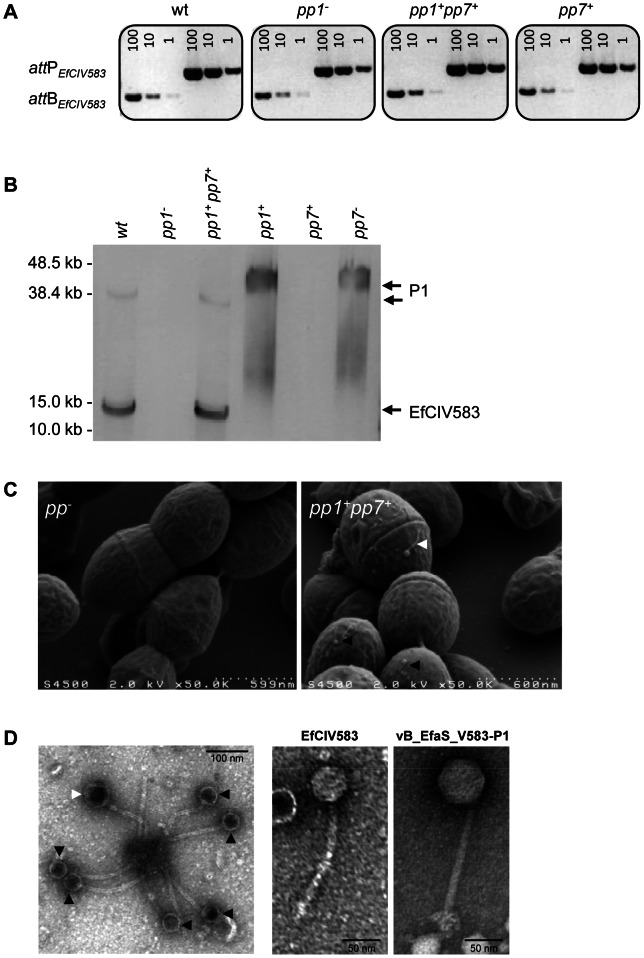
Interaction between *E. faecalis* pp1 and pp7 (EfCIV583). (A) Semi-quantitative PCR detection of EfCIV583 circular forms (*attP*) and excision sites (*attB*) in wild-type (WT) and strains *pp1^−^*, *pp1^+^ pp7^+^* and *pp7^+^*. Excision and circularization products probed by semi-quantitative PCR on 100, 10 and 1 pg of total bacterial DNA prepared from cultures of WT and strains *pp1^−^*, *pp1^+^ pp7^+^* and *pp7^+^* induced for 2 h with 2 µg/ml of ciprofloxacin at 37°C. Twenty cycles were used to amplify products of pp1 and EfCIV583. These results are representative of two independent experiments. (B) Prophage DNA extracted from precipitated phage particles obtained from lysates of WT and strains *pp1^−^*, *pp1* ^+^ *pp7* ^+^ and *pp7* ^+^ was separated by FIGE and analyzed by Southern-blot and hybridized sequentially using specific probes for pp1 and EfCIV583 genomes. The approximately 38.2 kb and 12 kb band corresponds to P1 and EfCIV583 genome, respectively. As ascertained by pp1-specific hybridization, migration of P1 DNA was delayed in lane *pp1* ^+^ and *pp7* ^−^ compared to lanes WT and *pp1* ^+^ *pp7* ^+^. Lambda DNA mono-cut mix (NEB) was run next to the samples to validate band sizes. (C) Scanning electron microscopy images of bacterial cells from strains *pp^−^* and *pp1* ^+^ *pp7* ^+^ after ciprofloxacin treatment. (D) Transmission electron microscopy images of phages produced by strain *pp1* ^+^ *pp7* ^+^ after ciprofloxacin treatment. White and black arrows indicate big and small sized particles attributed to P1 and EfCIV583, respectively. Enlarged images of EfCIV583 and P1 (renamed vB_EfaS_V583-P1) are shown on the right.

**Figure 5:**
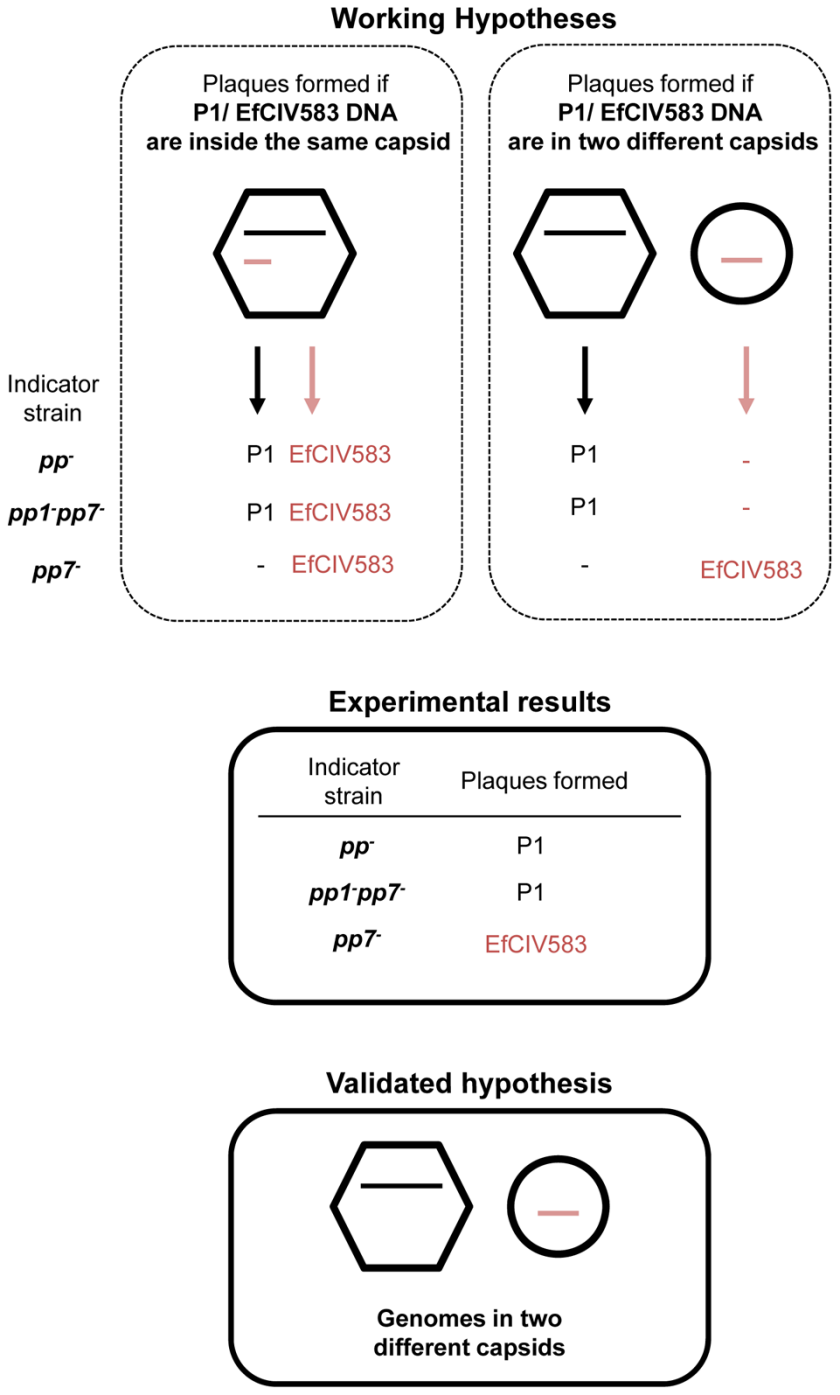
DNA of pp1 and EFCIV583 are packaged in separated capsids. Presentation of two working hypotheses for pp1 and EFCIV583 DNA packaging in a dilysogen strain and experimental results corroborating one of them. On the left, DNAs are packaged inside the same capsid. The resulting virions are predicted to deliver both DNA during infection and to form plaques containing both P1 and EFCIV583 virions since pp1 is required for formation of EFCIV583 virions on indicator strains *pp^−^* and *pp1* ^−^ *pp7* ^−^, both deleted for pp1 and EFCIV583. On the right, pp1 and EFCIV583 DNAs are packaged separately in two different capsids. The resulting virions would deliver either pp1 or EFCIV583 DNA during infection of strains *pp^−^* and *pp1* ^−^ *pp7* ^−^, and would form only P1 plaques since pp1 is required for formation of EFCIV583 virions and co-infection by two particles is highly improbable. However, EFCIV583 virions would be detected on the indicator strain *pp7^−^*, which harbors pp1. Lysates of strain *pp1^+^ pp7^+^* were tested on indicator strains *pp* ^−^, *pp1* ^−^ *pp7* ^−^ and *pp7^−^* and the resulting plaques were identified by pp1- and EFCIV583-specific PCRs. Our results strongly support that P1 and EfCIV583 genomes are packaged in two different capsids since plaques formed by *pp1^+^ pp7^+^* lysates on indicator strains *pp^−^* and *pp1^−^ pp7^−^* were identified as P1 plaques only, while EFCIV583 virions were detected on indicator strain *pp7^−^*.

To identify the step at which pp1 was required for production of EfCIV583 virions, we analyzed both the excision and replication of EfCIV583 and the packaging of EfCIV583 DNA in WT and the isogenic strains *pp1* ^−^, *pp1* ^+^ *pp7* ^+^, *pp7* ^+^ and *pp1* ^+^ by semi-quantitive PCR. Excision (*attB* region) and replication (*attP* region) products of EfCIV583 were detected in *pp1* ^−^ and *pp7* ^+^ strains at the same level as strains wild type and *pp1* ^+^ *pp7* ^+^ (Figure 4A), showing that pp1 is not required for EfCIV583 excision and replication. Next, DNA from phage particles produced by ciprofloxacin-treated WT and the isogenic strains *pp1* ^−^, *pp1* ^+^ *pp7* ^+^, *pp7* ^+^ and *pp1* ^+^ was recovered and analyzed as described above. Particles containing EfCIV583 DNA were recovered from WT and *pp1* ^+^ *pp7* ^+^ strains, while EfCIV583 DNA was no longer packaged in the absence of pp1 (strain *pp1* ^−^) or when present as a single element (strain *pp7* ^+^) (Figure 4B), indicating that pp1 is required for packaging of EfCIV583 DNA. Independent hybridizations revealed that EfCIV583 DNA is encapsidated as monomers only since no signal was detected at high molecular weight (data not shown). Noticeably, while the amount of the EfCIV583 DNA was similar between strains, the amount of pp1 DNA increased significantly when EfCIV583 was deleted (strain *pp1* ^+^), suggesting that EfCIV583 DNA hijacks P1 proteins at the expense of P1 particles production. The above molecular evidences for EfCIV583 pirating P1 proteins correlate with respective phage titers (Table 3). First, EfCIV583 titer was 10-fold higher than the titer of P1 in lysates from strain *pp1* ^+^ *pp7* ^+^, supporting that when present, EfCIV583 outnumbers P1 particles. Secondly, P1 titer of lysates from strain *pp1* ^+^ was 100-fold higher than in lysates from strain *pp1* ^+^ *pp7* ^+^, indicating that EfCIV583 impairs the production of P1 particles. Interestingly, P1 particles are infectious on strains *pp1* ^−^ *pp7* ^−^ and *pp* ^−^, but not on strains *pp1* ^−^ nor *pp7^+^* (Figure 5 and Table 3), further supporting that EfCIV583 interferes with P1 growth.

**Table 3:** Production of infectious P1 and EfCIV583 virions.

Indicator strain	Lysate (pfu/ml)		
	WT	*pp1^+^ pp7^+^*	*pp1^+^*
*pp^−^*	3.0×10^2^	1.6×10^3^	1.1×10^5^
*pp1^−^ pp7^−^*	1.5×10^2^	4.0×10^3^	1.5×10^5^
*pp1^−^*	-	-	-
*pp7^−^*	2.0×10^3^	1.8×10^4^	-

SaPI are usually encapsidated into small headed phage particles, distinguishable from their helper phage particles [29]. Indeed here, as pp1 and EfCIV583 genomes differ in size, P1 and EfCIV583 particles were expected to be distinguishable in size. Scanning electron microscopy observation of a ciprofloxacin treated culture from the *pp1^+^ pp7^+^* dilysogen revealed the existence of two phage size particles (Figure 4C), which were further confirmed by transmission electron microscopy (Figure 4D). Measurement of the capsids grouped the particles into small and large-size groups of ∼46 nm and ∼62 nm of width, respectively (Figure S2). Both particles harbored similar size tail of ∼165 nm in length. As a control, P1 particles obtained from strain *pp1^+^* were also analyzed. Their size corresponds to that of the large-size capsids produced by strain *pp1^+^ pp7^+^*, strongly indicating that large and small capsids belong to P1 and EfCIV583 virions, respectively. In addition, we confirmed that P1 belongs to the *Siphoviridae* family with a non-contractile tail (Figure 4D). Accordingly to Kropinsky's nomenclature proposal for bacterial virus [57], we propose to rename phage 1 “vB_EfaS_V583-P1”.

During completion of this manuscript, Duerkop et al, proposed that P1 and EfCIV583 were encapsidated together in a composite phage [58]. This hypothesis does not fit with the results reported here, and to completely exclude this possibility, we investigated particles infectivity on different indicator strains and identified the resulting plaques by phage-specific PCR (Figure 5). A mixed lysate of P1 and EfCIV583 was propagated on strains devoid of both pp1 and EfCIV583 (e. g. strains *pp* ^−^ and *pp1* ^−^ *pp7* ^−^). Under such circumstance, EfCIV583 should not form plaques, unless its DNA is indeed always encapsidated with that of its helper phage (Figure 5). Plaques were screened for the presence of EfCIV583 DNA, and none were found positive. As a control, the same lysate grown on a pp1 positive lawn gave EfCIV583 positive plaques, as expected. Thus, we conclude that EfCIV583 DNA is encapsidated separately into small size particles, and does not travel along with its helper phage. We can nevertheless explain how Duerktop et al. came to their inappropriate conclusion (see discussion). According to our results and in keeping with SaPI elements, pp1 and EfCIV583 DNA are packaged in distinct particles and we propose that large and small phages correspond to packaging of pp1 and EfCIV583 DNA, respectively. Altogether, our results demonstrate that EfCIV583 is a self-excisable and -replicative phage-related element, using P1 as a helper phage.

### Prophage 1 interferes with pp4 excision

Having observed that pp4 excises spontaneously at 37°C in a monolysogen strain, we analyzed the presence of pp4 circular forms in a panel of strains containing various prophages to understand which one(s) was interfering with its excision (Figure S3). Interestingly, the presence of pp4 circular forms at 37°C was strictly correlated with the absence of pp1 prophage, indicating that pp4 excision is blocked at 37°C when pp1 is present. Spontaneous excision of pp4 at 42°C in WT strain suggests that the inhibitory effect of pp1 is thermosensitive. Indeed, P1 titer of supernatants of a monolysogen strain increased 10-fold, from 10^4^ pfu/ml to 10^5^ pfu/ml when grown at 28 and 42°C, respectively, supporting that P1 repressor is thermosensitive. This result reveals another level of *E. faecalis* prophage interactions, in which prophage pp1 interferes with excision of prophage pp4.

### Potential plasmid-prophage interactions

We next investigated whether phages were produced as readily in the V583 parental strain as in the plasmid-cured strain used hitherto. For this, supernatants of V583 cultures treated or not with ciprofloxacin were plated on the same set of indicator strains *pp1^−^*, *pp3^−^*, *pp4^−^*, *pp5^−^*, *pp6^−^* and *pp7^−^* as above. PRCI EfCIV583, but not P3 nor P5 was found to form plaques on the corresponding deleted strains. These results show that strain V583 exhibits a lower efficiency of phage production compared to its plasmid-cured derivative, and suggest that plasmid-curing has somehow caused an increase of the basal level of prophage induction, indicating a possible interference of plasmids with prophages. Since plasmid pCM194 can increase phage production of a SPO2 lysogen *Bacillus subtilis* strain [59], it is also possible that V583 plasmids interfere negatively with phage production and contribute to prophage accumulation leading to polylysogenic strains.

### Impact of V583 prophages on *E. faecalis* biological traits

Infectious virions produced by lysogenic strains are likely to form progeny on phage sensitive strains and thereby may provide a selective advantage in a complex ecosystem. Since P1 and EfCIV583 are the most efficiently produced and are enriched in CC2 isolates, we evaluated the role of *E. faecalis* pp1 and EfCIV583 in the colonization of the mouse gastro-intestinal tract (GIT). We compared the ability of strains WT and *pp1^−^*, which no longer produces P1 and EfCIV583, to colonize separately the GIT of clindamycin-treated mice for 4 days (Figure S4). No significant difference in the efficiency of the GIT colonization was observed between the WT and the *pp1^−^* strains, indicating that either the presence of pp1 or the production of P1 and EfCIV583 virions is dispensable for GIT colonization by *E. faecalis* V583 in a complex ecosystem of intestinal microbiota. Further work, as testing the two strains in competition for instance, is needed to detect more subtle effects, as recently reported in a simple ecosystem [58].

**Figure 6:**
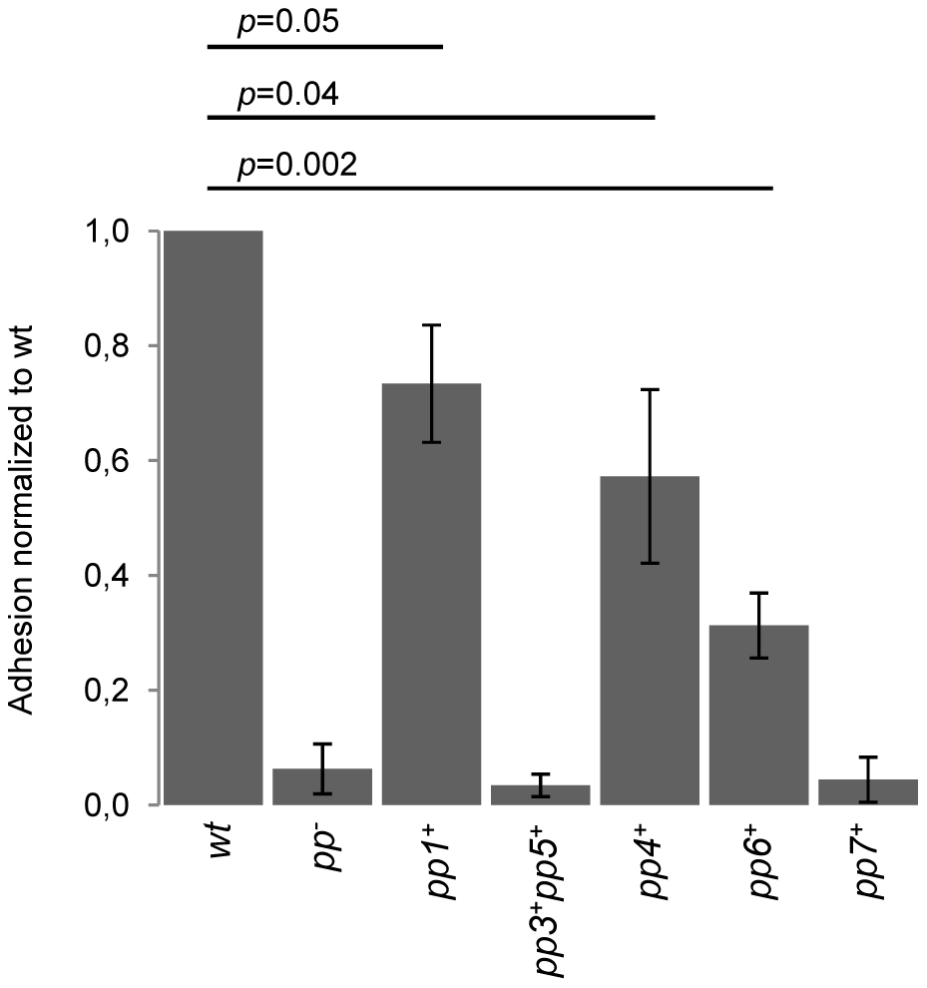
Impact of prophages on *E. faecalis* adhesion to human platelets. The values shown are normalized to the percentage of adhesion to platelets of the WT strain. Data are expressed as mean ± SD. Platelet binding assays were performed in platelets from three different donors. P value between WT strain and adherent strains (*pp1^+^*, *pp4^+^* and *pp6^+^*) is indicated.

Prediction of prophage-encoded platelet-binding factors also prompted us to investigate *E. faecalis* V583 prophages impact on binding to human platelets. Since neither pp3 nor pp5 encode predicted Pbl, strain *pp3^+^ pp5^+^* that harbors pp3 and pp5 only, was constructed as a negative control and verified for the production of P3 and P5 particles (Table S1). We tested the ability of strains WT, *pp^−^*, *pp1^+^*, *pp4^+^*, *pp6^+^*, *pp7^+^* and *pp3^+^ pp5^+^* to bind human platelets (Figure 6). Removal of six V583 prophages (strain *pp^−^*) reduced by ∼8-fold the adhesion ability of *E. faecalis*, revealing that prophages contribute to the interaction with human platelets. Similarly to *pp^−^* strain, strains that have pp3 and pp5 or EfCIV583 bound poorly to platelets, indicating that pp3, pp5 and pp7 are not involved in platelet adhesion. In contrast, strains carrying prophage-encoding platelet-binding factors, i.e. *pp1* ^+^, *pp4* ^+^ and *pp6* ^+^, bound significantly more than *pp^−^* strain, with a significant higher platelet adhesion for strains *pp1^+^* and *pp4^+^*. These results show that *E. faecalis* platelet-binding ability correlates with prophage-encoding platelet-binding factors. Given the importance of bacterial-platelet binding in the development of infective endocarditis it is tempting to speculate that these phages might contribute to enrich the repertoire of virulence traits of the species.

## Discussion

In the present study, we characterized the biological activity (excision, replication and virion production) of six *E. faecalis* predicted prophages of a plasmid-cured derivative of the polylysogenic V583 isolate, a representative of the hospital-adapted clade CC2. We show that all of the predicted prophages, except V583-pp2, are able to start a lytic cycle, with four of them (V583-pp1, V583-pp3, V583-pp5 and V583-pp7) leading to the production of phage progeny, which is exacerbated by clinically relevant antibiotics. Besides showing that phages P1, P3 and P5 are autonomous and confer self immunity to their bacterial host, we identified three levels of prophage interactions: i) the herein demonstrated phage-related chromosomal island EfCIV583 (V583-pp7) hijacks P1 capsids and interferes with P1 infectivity, ii) pp1 exerts a temperature-dependent inhibition of pp4 excision, and iii) pp3 and pp5 block excision of pp6. We also pinpoint three prophages that participate to *E. faecalis* V583 adhesion to human platelets, considered as a first step towards the development of infective endocarditis. Altogether, the interplay between these prophages potentiates their mobility and biological activities.

**Figure 7:**
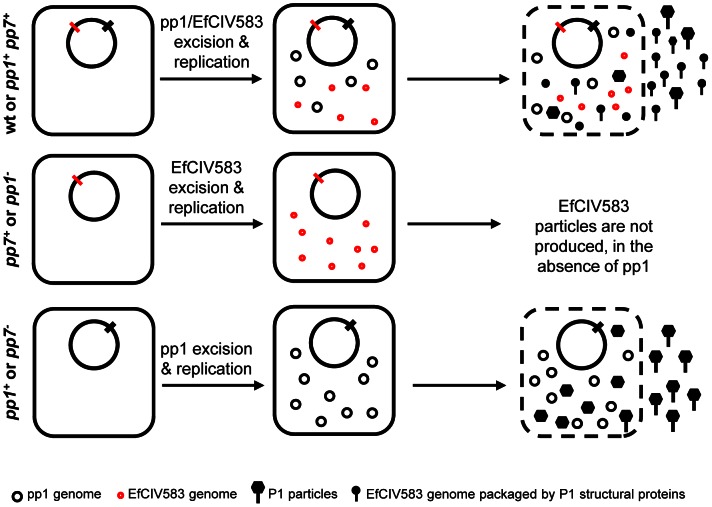
Model of P1/EfCIV583 interplay. Infectious P1 and EfCIV583 particles are produced by strains WT and *pp1^+^ pp7^+^* whereas no particles are produced in the absence of pp1 (strains *pp1^−^* and *pp7^+^*), showing that pp1 is required to form EfCIV583 virions. This hijacking phenomenon impairs the production of P1 particles in favor of EfCIV583. As observed by SEM and TEM, strain *pp1^+^ pp7^+^* produces two different sizes of phage particles: the biggest package most probably P1 DNA and the smallest EfCIV583 DNA. In the absence of EfCIV583 (strains *pp7^−^* and *pp1^+^*), P1 virions are produced at higher titer.

Polylysogeny is found in a variety of bacterial species, including *E. faecalis* [46], and it is frequently considered as the result of an adaptive evolution process in which prophages are maintained as they confer advantageous properties to the bacterial strains [35], [60]–[64]. As a way to maintain and propagate themselves, prophages interfere with each other through a variety of mechanisms in different bacterial species [31], [62], [65]. We demonstrate that EfCIV583 is a phage-related chromosomal island that excises and replicates autonomously as an episome, but specifically requires P1 structural proteins for production of infectious virions. Correlating the genome length of each prophage with the electron microscopic observations and virion infectivity, we propose that P1 and EfCIV583 virions encapsidate into large and small size particles, respectively. Moreover, as packaging of EfCIV583 DNA mobilizes P1 structural proteins, EfCIV583 outcompetes with the formation of P1 particles and interferes with P1 plaque forming ability. Our conclusions on P1 and EfCIV583 DNA packaging, and autonomy of the helper phage P1 differ from those recently reported by Duerkop *et al*., 2012 [58]. Their data can be fully explained by the chromosomal island-helper phage interaction that we have described between EfCIV583 and P1, except for the apparent absence of P1 particles in the supernatant of a V583 strain mutated for EfCIV583 (their Fig. 2D), which leads the authors to suggest that P1 depends on EfCIV583 for its growth. However, the indicator strains used in this experiment are not appropriate to count P1 plaques as they are lysogen for P1 and therefore immune to P1 (WTs of Fig. S6 in Duerkop *et al.*, 2012 [58]). According to our data and as depicted in Figure 7, the interaction between *E. faecalis* P1 and the phage-related chromosomal island EfCIV583 is a case of molecular piracy, which involves hijacking of P1 structural proteins by EfCIV583 DNA to be disseminated into small capsids. This is to our knowledge the first example of Gram positive, other than staphylococci, in which such molecular piracy phenomenon has been described. In spite of the resemblance with the well studied system of the SaPIs and their helper phages [26], [27], EfCIV583/P1 system is different in several ways. First, with the exception of SaPIbov1 and SaPIbov2 [66], SaPIs are generally stably maintained into the bacterial genome [26] through the action of a SaPI-encoded master repressor [28], which is inactivated by helper-phage specific antirepressors [67], [68]. Here, spontaneous excision of EfCIV583 in a monolysogen strain suggests that the activity of its predicted repressor (EF2954) is controlled by a helper phage-independent mechanism. Furthermore as EfCIV583 excision is increased by ciprofloxacin, this repressor is likely under the control of the SOS response. Similarly, SpyCIM1 of *S. pyogenes* responds to SOS system, however the implication of a helper phage for its induction remains to be investigated. Excision and reintegration of SpyCIM1 adjust the adaptation capacity of the host strain by modulating expression of the gene *mutL* [24], [69]. Given that EfCIV583 integrates into the promoter region of a putative xanthine/uracil permease gene, it may dynamically modulate xanthine or uracil utilization. Secondly, while the best studied SaPIs, SaPI1 and SaPIbov1, form mostly small capsids, their DNA can also be packaged as multimers into large capsids [70], [71]. In the case of EfCIV583, the packaging specificity seems to be tightly controlled since EfCIV583 DNA is packaged exclusively as the monomeric form. Lastly, different interference mechanisms used by the SaPIs to counteract capsid formation and DNA packaging of the helper phage have been recently deciphered [31], [32]. They rely on capsid morphogenesis (*cpm*) and phage packaging interference (*ppi*) genes of which no close homolog can be identified in EfCIV583 (R. Guerois, Pers. Comm.), suggesting that EfCIV583 may use other mechanisms to interfere with P1. Thus, besides expanding and strengthening the concept of molecular piracy within bacteria, the enterococcal EfCIV583/P1 system exhibits specific molecular mechanisms that deserve to be further investigated.

Remarkably, pp4 and pp6 are kept silent by other prophages. Prophage pp1 negatively interferes with pp4 excision at 37°C but not at 42°C. As excision of pp1 is increased at 42°C, a simple explanation may be that the pp1 repressor is itself thermosensitive and controls pp4. We also found that pp6 excises only when pp3 and pp5 are deleted from the wild type strain. Since single deletion of pp3 or pp5 had no effect on pp6 induction, it is conceivable that pp3 and pp5 exert redundant repression of pp6 induction. Noticeably, pp3 and pp5 share the highest homology compared to the other V583 prophages, suggesting a potential crosstalk. A recent study from Lemire *et al*., 2011 described a mechanism of antirepressor-mediated control of prophage induction involving recognition of both cognate and non-cognate repressors of Gifsy prophages in *Salmonella* [55]. Interestingly, their work suggests coordinate induction of lytic cycle of prophages in polylysogenic strains. Prophage interferences and low efficiency of V583 lysis upon induction may contribute to maintain diversity within the bacterial population and ensure survival. Further genetic and molecular studies will be required to characterize the crosstalk mechanisms between *E. faecalis* prophages, including prophage-related chromosomal islands.

Prophages are usually maintained in the host cell by mechanisms that block induction of the lytic cycle [72]. However, exposure to SOS-inducing signals such as DNA-damaging agents, reactive oxygen species or antibiotics can trigger lytic cycle [73]. In some cases, prophage induction provides a competitive advantage to the rest of the population in which the prophage is not induced, by activating expression of fitness and/or virulence genes that are associated with phages. For example, platelet binding activity of *S. mitis* is linked to lysis induced by phage SM1, implying that lytic activity of this prophage is required [19], [74]. Upon prophage induction, platelet binding proteins PblA and PblB, coded in φSM1, exert a dual function. They are part of φSM1 capsids as a tape measure and side tail fiber respectively, and they bind as free proteins to the cell wall of non-induced bacteria, allowing the bacterium to interact with platelets [74]. We provide for the first time direct evidence that pp1, pp4 and pp6 are important for *E. faecalis* adhesion to human platelets. Given that pp1, pp4 and pp6 encode predicted phage tail proteins homologous to platelet binding proteins PblA and/or PblB of φSM1 [43], [46], these proteins (namely EF0348, EF2003, EF2001, EF2811, EF2813) are likely to mediate *E. faecalis* binding to platelets. Interestingly, *E. faecalis* platelet-binding capacity varies from strong for the *pp1^+^* strain, to intermediate for the *pp4^+^* strain and to low for the *pp6^+^* strain. It is possible that the various platelet efficiencies are the direct consequence of Pbls distinct binding capacity. It should be noted however that such variation correlates with the efficiency of pp1, pp4 and pp6 to perform their lytic cycle. Indeed, adhesion is minimal for the strain that harbors pp6, which only excises, maximal for strain that forms pp1 progeny, and intermediate for that strain has pp4, which excises and replicates. Even if pp4 and pp6 don't form infectious virions, they excise from the chromosome suggesting that their genes are expressed. Thus, they are likely to impact on host biology. We propose that this correlation may reflect different levels of expression and/or accessibility resulting from prophage activity. Since adhesion to platelets can lead to platelet activation, which promotes infective endocarditis, the role of prophage-encoded Pbl-like proteins in *E. faecalis* pathogenesis deserves further investigation. In addition to a PblA-like protein, pp1 encodes another protein with putative dual function: EFV toxin, a predicted minor head protein with an ADP-ribosyltransferase activity, which causes cell death upon heterologous expression in yeast [47]. Although toxin localization and molecular targets have yet to be discovered, the possibility exists that pp1 and EfCIV583 particles (that are made of P1 structural proteins), enhance the effect of ADP-ribosyltransferase activity.

Prophage excision also restores gene function or modify gene expression [17], [24]. In the case of *E. faecalis* V583 prophages, excision of pp4 restores an open reading frame of an operon that encodes orthologs of a DNA uptake machinery [54]. Interestingly, Rabinovitch *et al*., 2012 recently reported that excision of the *L. monocytogenes* prophage φ10403S induces the expression of a DNA uptake machinery needed for intracellular growth [17]. Although, probably not functional in strain V583, due to a premature stop codon in another gene of the same operon [54], such a complex is likely to be expressed in strains devoid of mutation. Resemblance of the DNA uptake machinery with type IV secretion systems suggests that its expression may confer new biological traits.

Gene dissemination is another important biological aspect through which temperate phages impact on bacterial species [30], [75], [76]. Prophage mediated gene transduction has been recently reported between *E. faecalis* strains and between enterococcal species [46], [77]. We demonstrated that V583 pp1, pp3, pp5 and EfCIV583 form infectious particles suggesting that they are capable of mediating horizontal gene transfer. Their excision is enhanced by SOS-triggering agents, including mitomycin C, trimethoprim and the fluoroquinolone antibiotic ciprofloxacin. Fluoroquinolones inhibit DNA gyrase and topoisomerase IV, and cause DNA double-strand breaks, as such, they are among the most efficient phage-inducing antibiotics [78], [79]. Fluoroquinolones promote release of Shiga toxins encoded by prophages from *Escherichia coli* [80], potentiate the spread of virulence traits in *Staphylococcus aureus* [81], and eventually reduce strain competitiveness [82]. Though the impact of the *E. faecalis* prophages in promoting both strain fitness and horizontal gene spreading has yet to be studied, phage-inducing antibiotics may contribute to the emergence of *E. faecalis* polylysogenic strains, such as V583. Treatment with fluoroquinolones was identified as a risk factor for infection or colonization by vancomycin-resistant enterococci in the U.S, where the CC2 isolates have emerged [83]. Bacteria may use their prophages as weapons to kill sensitive competitors and thereby colonize a niche [36], [84]. Whereas pp1 and EfCIV583 have no detectable effect on the ability of *E. faecalis* to colonize a complex ecosystem such as mouse microbiota treated with clindamycin, Duerkop *et al*. 2012 reported recently that pp1 and EfCIV583 confer competitive fitness against closely related strains in the intestine of monoxenic mice devoid of endogenous microbiota [58]. Considering that fluoroquinolones enhance V583 phages activity, these antibiotics may contribute to strain fitness in the gastro-intestinal tract.

Given the complexity of the interplay between V583 prophages, we anticipate that mixed *E. faecalis* subpopulations may be formed upon prophage induction and could favor survival of one or several of them as described for different bacterial species especially in biofilms [33], [85]–[87]. In all, temperate phages are likely to potentiate *E. faecalis* genetic and physiological flexibility for optimal adaptation during colonization or infection.

## Materials and Methods

### Bacterial strains and growth conditions

Strains used in this study are listed in Table S1. *E. coli* strains were grown at 37°C in LB medium with shaking. *E. faecalis* strains were grown in static conditions in appropriate media, either BHI or M17 supplemented with 0.5% glucose (M17G) at 37°C, unless differently stated. Growth was monitored by measuring optical density at 600 nm (OD_600_). Antibiotics were used at the following concentrations: erythromycin, 10 µg/ml for *E. faecalis* and 150 µg/ml for *E. coli*; and ampicillin 100 µg/ml.

### Prophage induction, total DNA extraction and semi-quantitative PCR

*E. faecalis* strains were grown at 37°C in M17G up to OD_600 = _0.2, and prophages were induced by adding mitomycin C, ciprofloxacin, trimethoprim or ampicillin at a final concentration of 4 µg/ml, 2 µg/ml, 0.04 µg/ml and 2 µg/ml, respectively. Cultures were grown for 2 hours at 28, 37 or 42°C, depending on the experimental assay. Cells were collected by centrifugation at 4°C and total DNA was extracted as previously [53]. The resulting DNA samples were screened for circular form of phage DNA (*attP* region) and for the excision site left on the chromosome (*attB* region) after prophage induction, by PCR using primer pairs (Table S2): ef303f/ef0355f and ef0302f/ef0357f (pp1), OEF591/OEF592 (pp2), OEF531/OEF532 and OEF653/OEF654 (pp3), OEF546/OEF547 and OEF551/OEF640 (pp4), OEF533/OEF534 and OEF655/OEF656 (pp5), OEF548/OEF549 and OEF557/OEF624 (pp6) and OEF560/OEF561 and OEF585/OEF657 (pp7). Control PCR was performed with primers targeting the chromosomal gene *ef3155* using ef3155f and ef3155r primers, listed in Table S2. PCR amplifications were carried out in a Mastercycler gradient apparatus (Eppendorf, Courtaboeuf, France) using Taq DNA polymerase (Qbiogene, Illkirch, France). Analysis of PCR products was monitored by agarose gel electrophoresis. Semi-quantitative PCR was performed on serial dilutions of quantified DNA recovered from both induced and uninduced cultures. For each DNA sample, both *attB* and *attP* regions were amplified using from 20 to 32 cycles. Excision *versus* replication was evaluated by comparing the amount of *attB* and *attP* PCR products, respectively, after gel electrophoresis. For each prophage, attachment site sequence was determined by sequencing the PCR products corresponding to the junction of both the circular form and the excision site. Sequencing was performed by GATC Biotech, Germany.

### Construction of prophage deleted strains

Independent markerless deletions on different phage combinations were constructed through double-crossing over as described previously [88]. The 5′- and 3′-terminal regions of each phage were PCR-amplified from V583 chromosomal DNA and fused by PCR such that the attachment site (*att*B) was reconstructed allowing for re-infection by the cognate phage. PCR amplifications were made with the following primers: OEF634/OEF635 and OEF636/OEF637 (pp1), OEF470/OEF471 and OEF472/OEF473 (pp3), OEF626/OEF627 and OEF628/OEF629 (pp4), OEF476/OEF477 and OEF478/OEF479 (pp5), OEF618/OEF619 and OEF620/OEF621 (pp6), OEF641/OEF642 and OEF643/OEF644 (pp7), listed in Table S2. All the plasmids obtained during this work are listed in Table S1. The strain deleted for all studied prophages (strain *pp^−^*) was obtained by removing prophages 3, 5, 4, 6, 7 and 1 sequentially. Sequencing of the deletion site and PFGE confirmed the deletion and the absence of other major genome rearrangements.

### Phage DNA extraction, field-inverted gel electrophoresis (FIGE) and Southern-blot

V583 prophages were induced by addition of ciprofloxacin at 2 µg/ml to 100 ml of an exponential-phase culture (OD_600_∼0.2) further cultivated for 4 h at 28, 37 and 42°C. The induced culture was centrifuged at 6 500 g for 20 minutes at 4°C. The supernatant was collected and filtered through a 0.22 µm filter. Filtrate was supplemented with PEG 6000 (10% final conc., v/v) and NaCl 1 M and incubated overnight at 4°C. Phage particles were then pelleted by centrifugation at 7 600 g for 1 hour at 4°C. Supernatant was discarded and the phage pellet was soaked. The pellet was resuspended in 100 µl of SM buffer [89]. PEG was removed by chloroform extraction before treating the phage particles with 4 units of DNase I (Sigma) for 1 hour at 37°C to remove contaminating bacterial chromosomal DNA. Next, phage particles were disrupted at 80°C for 10 minutes in the presence of SDS 1%, proteins were removed by phenol/chloroform extraction, DNA was precipitated with ethanol and finally resuspended in 20 µl of TE containing 20 µg/ml of DNAse-free RNaseA (Sigma). DNA from phage particles was analyzed by field inversion gel electrophoresis (FIGE, BioRad) on 1% agarose gel in TBE for 22 h at 11°C. Migration conditions were the following: forward voltage 6 V/cm, reverse voltage 4 V/cm, switch time 0.2–1.0 sec, linear ramp. The gel was stained with ethidium bromide and monitored on a UV transillumination table, before transferring DNA onto a Nylon membrane (QBiogene) by Southern-blot [53]. Individual phage genomes were identified by hybridization with phage-specific probes amplified by PCR on genomic DNA with the following primers (Table S2): OEF573/OEF574 (pp1); OEF575/OEF576 (pp3); OEF577/OEF578 (pp4); OEF488/OEF489 (pp5); OEF579/OEF580 (pp6); OEF581/OEF582 (pp7). Probe labelling and hybridization detection was performed with DIG DNA labeling and detection kit (Roche) according to manufacturer's instructions.

### Phage lysis plaque assay

Two ml of ciprofloxacin phage-induced cultures were collected after centrifugation for 20 min at 6 000 g at 4°C. Supernatants were collected and filtered on 0.22 µm filters. Filtrates were tested on indicator strain for plaque formation. Briefly, 50 µl of indicator strain grown in BHI up to OD_600_∼0.2 was mixed with 4 ml of BHI containing 0.2% agarose (Lonza, LE) and 10 mM MgSO_4_ and plated to form a lawn. 10 µl of each filtered-supernatant sample were spotted on the indicator bacterial lawn. Plates were incubated overnight at 28, 37 or 42°C. Plaque formation was visually detected. When needed, plaques were identified by PCR in two independent experiments. Briefly, twenty plaques formed on each indicator strains were probed systematically for both pp1 and EFCIV583 with specific primers (Table S2).

### Scanning electron microscopy (SEM)

Bacterial suspensions immersed in a fixative solution (2.5% glutaraldehyde in 0.2 M sodium cacodylate buffer, pH 7.4) were deposited on sterile cover-glass discs (Marienfeld, VWR, France) and kept 1 hour at room temperature before overnight storage at 4°C. The fixative was removed, and samples were rinsed three times for 10 min in the sodium cacodylate solution (pH 7.4). The samples underwent progressive dehydration by soaking in a graded series of ethanol (50 to 100%) before critical-point drying under CO_2_. Samples were mounted on aluminum stubs (10 mm diameter) with conductive silver paint and sputter coated with gold-palladium (Polaron SC7640; Elexience, France) for 200 s at 10 mA. Samples were visualized by field emission gun scanning electron microscopy. They were viewed as secondary electron images (2 kV) with a Hitachi S4500 instrument (Elexience, France). Scanning Electron Microscopy analyses were performed at the Microscopy and Imaging Platform MIMA2 (Micalis, B2HM, Massy, France) of the INRA research center of Jouy-en-Josas (France).

### Transmission electron microscopy (TEM)

Lysates of strains *pp1^+^* and *pp1^+^ pp7^+^* were recovered 6 h after ciprofloxacin-induction at 2 µl/ml. P1 was propagated on *pp^−^* strain and EfCIV583 on *pp1^+^* as described previously for phage plaque assay. Phages were recovered from the top agarose by addition of water and diffusion at 4°C during 4 hours. Next, samples were centrifuged for 15 minutes at 7 000 g and supernatant was filtrated through 0.22 µm pore filters. Phages samples were concentrated by ultra filtration through a Centricon YM-100 filter unit (Millipore, Molsheim, France). Bacteriophage solutions were applied on carbon-coated grids and subsequently stained with uranyl acetate (2% in water). Observations were performed with a JEOL 1200 EXII electron microscope.

### Mouse intestinal colonization model

Six to 8-weeks old male CF-1 mice (Harlan, USA) were used for intestinal colonization experiments as described previously [90]. Briefly, mice received a daily dose of 1.4 mg of subcutaneous clindamycin for three days. On the fourth day, mice were force-fed with 10^10^ colony-forming units (CFU) of *E. faecalis* strains prepared as dried frozen pellets as described previously [53]. Stool samples were collected at baseline and at 1 and 4 days after inoculation of the strains. The *E. faecalis* strains in feces sample were detected by plating diluted stool samples onto BEA supplemented with vancomycin at 6 µg/mL to monitor the inoculated strain. All animals were handle in strict accordance with good animal practice as defined by the local animal welfare bodies (Unité IERP, INRA Jouy-en-Josas, France) and all animal work were carried out under the authority of license issued by the Direction des Service Vétérinaires (accreditation number A78-187 to LR-G).

### Platelet binding assay

The ability of *E. faecalis* to bind to human platelets was assessed as previously described [91]. Briefly, platelets-enriched cells were washed, fixed in paraformaldehyde 3.2% and immobilized on poly-L-lysine for 1 h at 37°C. Unbounded platelets were washed with PBS prior to saturation with a 1% casein solution for 1 h at 37°C. After removal of the saturating solution, platelets were incubated for 1 h at 37°C with the indicated bacteria at a MOI of 1. After extensive washes, the numbers of bound bacteria were assessed by colony forming units (CFUs). Binding was expressed as a percentage of the inoculum followed by normalization to wild-type strain adhesion. Platelet binding assays were performed three times in triplicate using platetelets prepared from buffy coats of three, healthy and anonymous volunteers obtained through the Etablissement français du sang (EFS, Ile de France, Le Chesnay, France). As required for blood donation, written informed consents were obtained by EFS from all donors.

## Supporting Information

Figure S1 **Growth of ciprofloxacin-treated strains.** The WT and isogenic strains *pp^−^*, *pp1^+^* and *pp3^+^ pp5^+^* were grown at early exponential growth phase (OD_600_∼0.2) before treatment with ciprofloxacin at 4 or 6 µg/ml. Relative optical density (OD_600_) was calculated for each strain as the ratio of OD_600_ of the ciprofloxacin-induced cultures (Cip 4 and Cip 6) with the non-induced culture (Cip 0) 6 h after addition of ciprofloxacin later. The mean and the standard error of the mean (SEM) obtained on two independent cultures for each strain is shown.
(TIF)

Figure S2 **Capsid size distribution of virions produced by strains** ***pp1^+^ pp7^+^*** **and** ***pp1^+^*** **.** Scatter plot of the capsid width (nm) measured particles for strains *pp1^+^ pp7^+^* (n = 42) and *pp1^+^* (n = 24). Strain *pp1^+^ pp7^+^* produced two groups of different capsid size, small and large with a mean width of 46.1±3.7 nm and 61.1±1.3 nm, respectively. Strain *pp1^+^* produced homogenous capsid size with a mean width of 62.9±3.3 nm.
(TIF)

Figure S3 **pp1 interference with pp4 excision.** PCR detection of pp4 circular forms in different isogenic strains (see Figure 2A): WT, *pp3^−^*, *pp3^−^ pp5^−^*, *pp1^−^ pp3^−^ pp5^−^*, *pp4^+^ pp6^+^* and *pp4^+^* (see Table S1). Circular forms of pp4 are detected only in the absence of pp1.
(PDF)

Figure S4 **Mice gastro-intestinal tract colonization by strains WT and** ***pp1^−^*** **.** After three days of subcutaneous administration of clindamycin, 1×10^10^ CFUs of each strain (WT or *pp1^−^*) were force-fed in five mice. *E. faecalis* burden in stools was monitored daily for four days after oral gavage. No significant differences in the efficiency of colonization between strains was observed.
(PDF)

Table S1 **Bacterial strains and plasmids used in this study.**
(DOC)

Table S2 **Primers used in this study.**
(DOC)

Table S3 **V583 phages infection and immunity.**
(DOC)
