# Supplementary figures and images for: Enterococcus faecalis Prophage Dynamics and Contributions to Pathogenic Traits
Source: PLoS Genet. 2013 Jun 6;9(6):e1003539. doi: 10.1371/journal.pgen.1003539 (PMC3675006; doi:10.1371/journal.pgen.1003539)

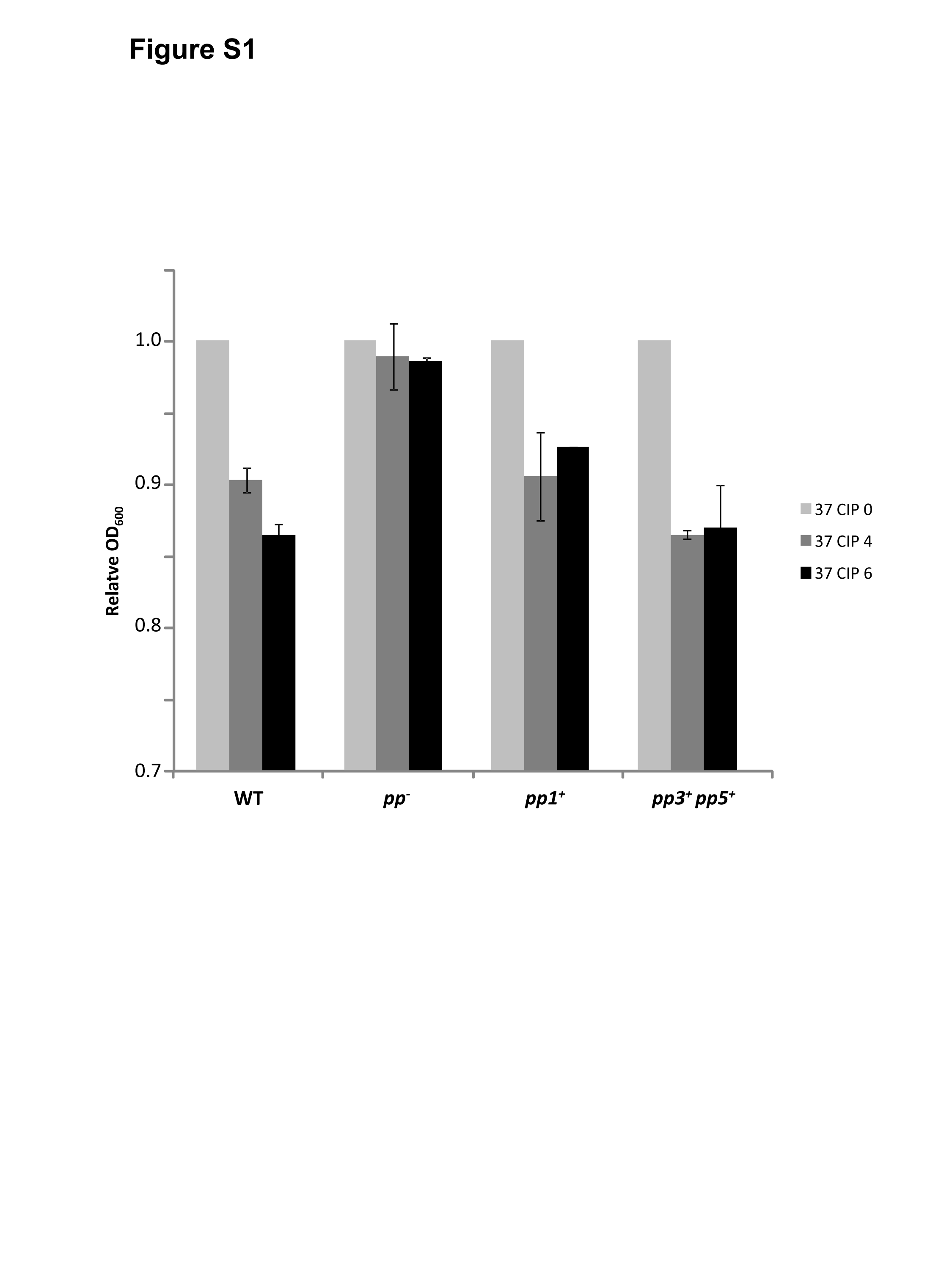

Supplement: Figure S1 — Growth of ciprofloxacin-treated strains. The WT and isogenic strains pp−, pp1+ and pp3+ pp5+ were grown at early exponential growth phase (OD600∼0.2) before treatment with ciprofloxacin at 4 or 6 µg/ml. Relative optical density (OD600) was calculated for each strain as the ratio of OD600 of the ciprofloxacin-induced cultures (Cip 4 and Cip 6) with the non-induced culture (Cip 0) 6 h after addition of ciprofloxacin later. The mean and the standard error of the mean (SEM) obtained on two independent cultures for each strain is shown. (TIF) [file pgen.1003539.s001.tif]

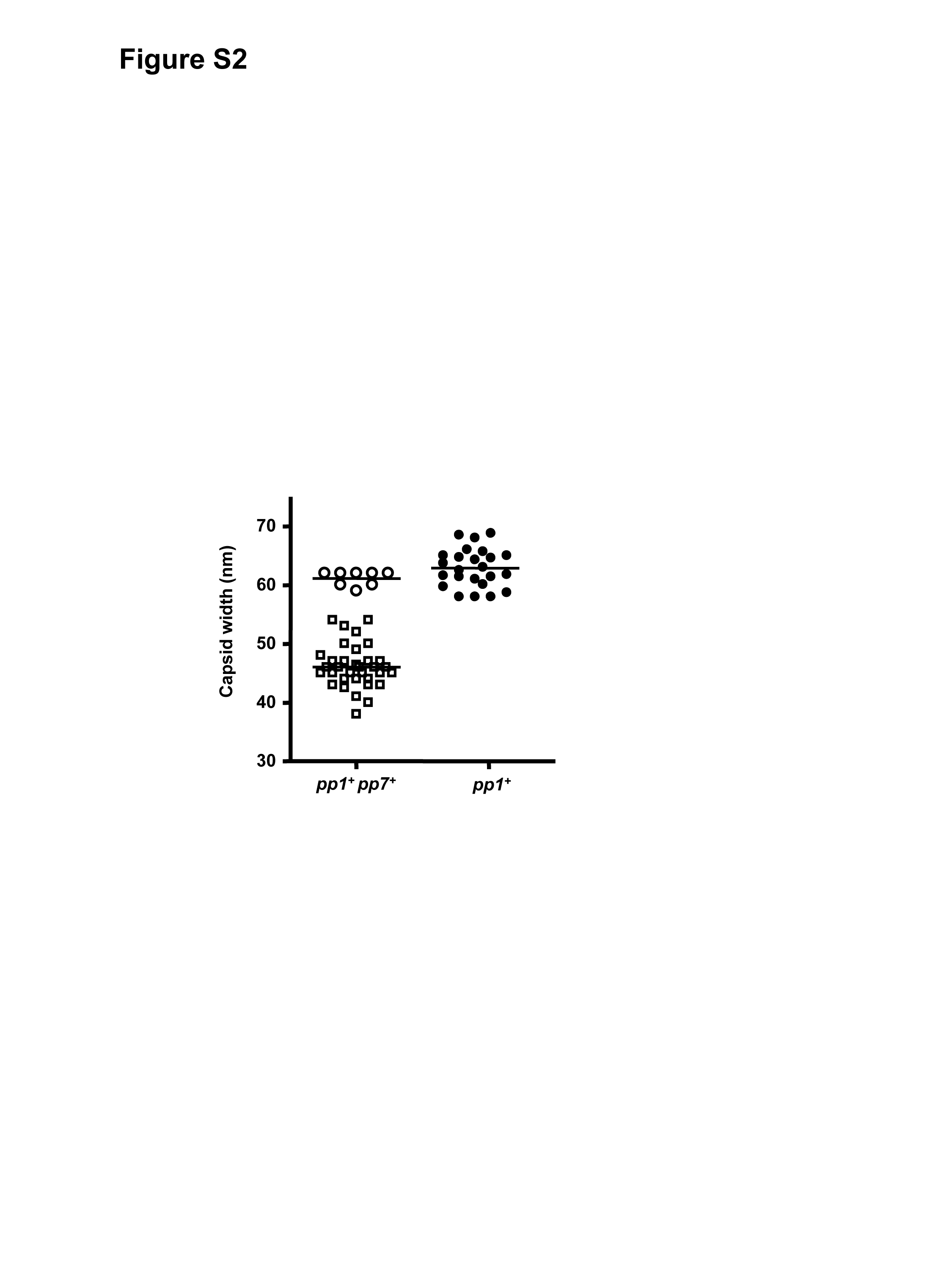

Supplement: Figure S2 — Capsid size distribution of virions produced by strains pp1+ pp7+ and pp1+ . Scatter plot of the capsid width (nm) measured particles for strains pp1+ pp7+ (n = 42) and pp1+ (n = 24). Strain pp1+ pp7+ produced two groups of different capsid size, small and large with a mean width of 46.1±3.7 nm and 61.1±1.3 nm, respectively. Strain pp1+ produced homogenous capsid size with a mean width of 62.9±3.3 nm. (TIF) [file pgen.1003539.s002.tif]

**Figure S3**

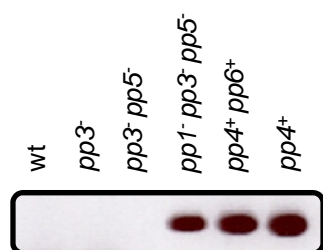

Supplement: Figure S3 — pp1 interference with pp4 excision. PCR detection of pp4 circular forms in different isogenic strains (see Figure 2A): WT, pp3−, pp3− pp5−, pp1− pp3− pp5−, pp4+ pp6+ and pp4+ (see Table S1). Circular forms of pp4 are detected only in the absence of pp1. (PDF) [file pgen.1003539.s003.pdf]

Figure S4

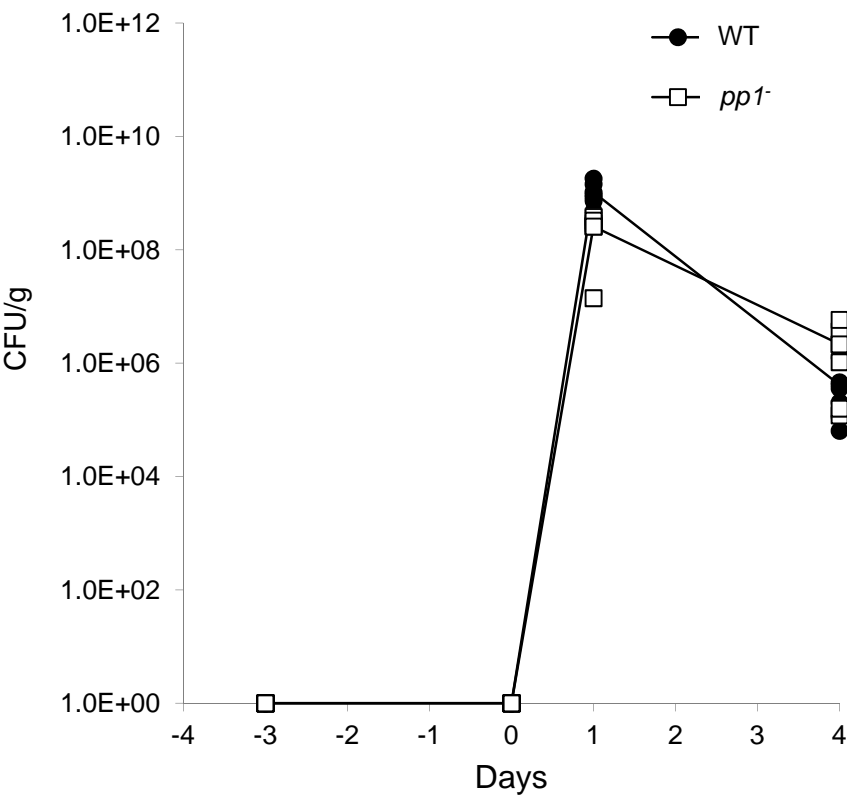

Supplement: Figure S4 — Mice gastro-intestinal tract colonization by strains WT and pp1− . After three days of subcutaneous administration of clindamycin, 1×10^10^ CFUs of each strain (WT or pp1−) were force-fed in five mice. E. faecalis burden in stools was monitored daily for four days after oral gavage. No significant differences in the efficiency of colonization between strains was observed. (PDF) [file pgen.1003539.s004.pdf]
