# Supplementary material for: Enterococcus faecalis Prophage Dynamics and Contributions to Pathogenic Traits
Source: PLoS Genet. 2013 Jun 6;9(6):e1003539. doi: 10.1371/journal.pgen.1003539 (PMC3675006; doi:10.1371/journal.pgen.1003539)
Supplement: Table S1 — Bacterial strains and plasmids used in this study. (DOC) [file pgen.1003539.s005.doc]

**Table S1**. Bacterial strains and plasmids used in this study.

| **Strain or plasmid** | **Short name** | **Relevant characteristics** | **Reference or source** |
| --- | --- | --- | --- |
| **Strain** |  |  |  |
| *E. coli* |  |  |  |
| TG1 |  | *supE hsdD5 thi* (∆*lac-proAB*) F’ (*traD36 proAB-lacZ∆M15*) |  |
| GM1674 |  | *dam^-^dcm^-^ repA^+^* |  |
| *E. faecalis* |  |  |  |
| VE14002 | V583 | V583 vancomycin resistant clinical isolate |  |
| VE14089 | WT | V583 vancomycin resistant clinical isolate cured of its plasmids |  |
| VE14279 | *pp3^ˉ^* | pp3 deletion in *E. faecalis* VE14089 | This study |
| VE18306 | *pp6^ˉ^* | pp6 deletion in *E. faecalis* VE14089 | This study |
| VE18310 | *pp4^ˉ^* | pp4 deletion in *E. faecalis* VE14089 | This study |
| VE18284 | *pp7^ˉ^* | pp7 deletion in *E. faecalis* VE14089 | This study |
| VE14285 | *pp5^ˉ^* | pp5 deletion in *E. faecalis* VE14089 | This study |
| VE18299 | *pp3^ˉ^ pp5^ˉ^* | pp3 and 5 deletion in *E. faecalis* VE14089 | This study |
| VE18313 | *pp1^ˉ^* | pp1 deletion in *E. faecalis* VE14089 | This study |
| VE18316 | *pp1^+^ pp7^+^* | pp3, pp4, pp5 and pp6 deletion in *E. faecalis* VE14089 | This study |
| VE18566 | *pp1^ˉ^ pp7^ˉ^* | pp1 and pp7 deletion in *E. faecalis* VE14089 | This study |
| VE18576 | *pp4^+^ pp6^+^* | pp1, pp3, pp5 and pp7 deletion in *E. faecalis* VE14089 | This study |
| VE18589 | *pp7^+^* | pp1, pp3, pp4, pp5 and pp6 deletion in *E. faecalis* VE14089 | This study |
| VE18590 | *ppˉ* | pp1, pp3, pp4, pp5, pp6 and pp7 deletion in *E. faecalis* VE14089 | This study |
| VE18559 | *pp1^ˉ^ pp3^ˉ^ pp5^ˉ^* | pp1, pp3 and pp5 deletion in *E. faecalis* VE14089 | This study |
| VE18562 | *pp1^+^* | pp3, pp4, pp5, pp6 and pp7 deletion in *E. faecalis* VE14089 | This study |
| VE18581 | *pp6^+^* | pp 1, pp3, pp4, pp5 and pp7 deletion in *E. faecalis* VE14089 | This study |
| VE18582 | *pp4^+^* | pp1, pp3, pp5, pp6 and pp7 deletion in *E. faecalis* VE14089 | This study |
| VE18583 | *pp3^+^ pp5^+^* | pp1, pp4, pp6 and pp7 deletion in *E. faecalis* VE14089 | This study |
| **Plasmids** |  |  |  |
| pGEM-T |  | Amp^r^, linearized with 3’ T overhangs, ori ColE1 | Promega |
| pG+host9 |  | Erm^r^, repATs |  |
| pVE14354 |  | Amp^r^, pGEM-T with *ef1417*-*ef1489* deletion | This study |
| pVE14357 |  | Amp^r^, pGEM-T with *ef2085*-*ef2145* deletion | This study |
| pVE14362 |  | Ery^r^, pG+host with *ef1417*-*ef1489* deletion | This study |
| pVE14364 |  | Ery^r^, pG+host with *ef2085*-*ef2145* deletion | This study |
| pVE14367 |  | Amp^r^, pGEM-T with *ef2798*-*ef2855* deletion | This study |
| pVE14368 |  | Ery^r^, pG+host with *ef2798*-*ef2855* deletion | This study |
| pVE14369 |  | Amp^r^, pGEM-T with *ef1988*-*ef2043* deletion | This study |
| pVE14370 |  | Ery^r^, pG+host with *ef1988*-*ef2043* deletion | This study |
| pVE14371 |  | Amp^r^, pGEM-T with *ef0303*-*ef0355* deletion | This study |
| pVE14372 |  | Ery^r^, pG+host with *ef0303*-*ef0355* deletion | This study |
| pVE13473 |  | Amp^r^, pGEM-T with *ef2936*-*ef2955* deletion | This study |
| pVE13474 |  | Ery^r^, pG+host with *ef2936*-*ef2955* deletion | This study |

1. Baer R, Bankier AT, Biggin MD, Deininger PL, Farrell PJ, et al. (1984) DNA sequence and expression of the B95-8 Epstein-Barr virus genome. Nature 310: 207-211.

2. Palmer BR, Marinus MG (1994) The *dam* and *dcm* strains of *Escherichia coli*--a review. Gene 143: 1-12.

3. Sahm DF, Kissinger J, Gilmore MS, Murray PR, Mulder R, et al. (1989) *In vitro* susceptibility studies of vancomycin-resistant *Enterococcus faecalis*. Antimicrob Agents Chemother 33: 1588-1591.

4. Rigottier-Gois L, Alberti A, Houel A, Taly JF, Palcy P, et al. (2011) Large-Scale Screening of a Targeted *Enterococcus faecalis* Mutant Library Identifies Envelope Fitness Factors. PLoS One 6: e29023.

5. Maguin E, Prevost H, Ehrlich SD, Gruss A (1996) Efficient insertional mutagenesis in lactococci and other gram-positive bacteria. J Bacteriol 178: 931-935.
