# Supplementary material for: Enterococcus faecalis Prophage Dynamics and Contributions to Pathogenic Traits
Source: PLoS Genet. 2013 Jun 6;9(6):e1003539. doi: 10.1371/journal.pgen.1003539 (PMC3675006; doi:10.1371/journal.pgen.1003539)
Supplement: Table S2 — Primers used in this study. (DOC) [file pgen.1003539.s006.doc]

**Table S2**. Primers used in this study.

| **Primer name** | **Sequence (5’→3’)*** | **Position of the primer 5’end^†^** | **Reference or source** |
| --- | --- | --- | --- |
| ef0302f | CGTGGATGGACGAATACAC | 289 146 | This study |
| ef0303f | GCAGTACAGATTATAAAA | 289 672 | This study |
| ef0355f | GATCGGCAACAAGTAATGTC | 326 034 | This study |
| ef0357f | AGAACATCAGTACATTTTACC | 326 635 | This study |
| ef3155f | ACAGCACCAGACCCGACAG | 3 026 754 | This study |
| ef3155r | ACGACGAGGTTCCATGTGATG | 3 026 256 | This study |
| OEF470 | AGACTAGAGTTACTACCCC | 2 003 732 | This study |
| OEF471 | AGCCACCTGCCGGGCTTG | 2 004 735 | This study |
| OEF472 | CAAGCCCGGCAGGTGGCTTTTTGGTAAAATCCCAACTC | 2 048 272 | This study |
| OEF473 | GCATCACCTGCGATATTTC | 2 049 399 | This study |
| OEF474 | TTGTGTTTAACACTCGGAGC | 2 003 577 | This study |
| OEF475 | AATTGGGTAGTTTCGACTTG | 2 049 708 | This study |
| OEF476 | GAAAGCGCCACAAATCTTC | 1 396 960 | This study |
| OEF477 | CTATAGGCGTGCATTTAAATC | 1 397 966 | This study |
| OEF478 | GATTTAAATGCACGCCTATAGTTTGTAAATAACAGAAGAAAAG | 1 446 359 | This study |
| OEF479 | AATAGAGATACGCTCAGCC | 1 447 403 | This study |
| OEF480 | CTGGCTGGATTGATTTACC | 1 396 770 | This study |
| OEF481 | CAACAGCTTCTTCACTCTC | 1 447 606 | This study |
| OEF483 | CATATTTTCACCTCCTATCC | 1 402 370 | This study |
| OEF485 | TCTCTAGGATGTTGTTGAGG | 1 401 513 | This study |
| OEF488 | GCCGCACATATAGATGATG | 2 045 368 | This study |
| OEF489 | CTAATTTCTTTGGTTCTGCC | 2 047 006 | This study |
| OEF490 | CAGAGTTCCTAGGAGTAAC | 2 045 792 | This study |
| OEF491 | GTTGTTCTAAGGTTGGCTC | 2 046 561 | This study |
| OEF531 | GACGTAGCAATGGTACTGG | 1 399 238 | This study |
| OEF532 | GATCGTTCAGGTAACATTAG | 1 445 898 | This study |
| OEF533 | ATCCGAAGGAACATTGCTAG | 2 005 292 | This study |
| OEF534 | CAATGGATTAACTGGCTTGC | 2 047 356 | This study |
| OEF546 | CAGTTCGAGTCCTGTATGG | 1 923 230 | This study |
| OEF547 | AGAACGGCTTTTCAGAGAAG | 1 962 161 | This study |
| OEF548 | ACCTAGCATTCGTAGAACC | 2 701 530 | This study |
| OEF549 | TACTATGCCGACTTAGACTG | 2 736 242 | This study |
| OEF551 | AGGCTACATTACTAAAGAGC | 1 962 985 | This study |
| OEF552 | TCCGTATTGATAAGCAATGC | 1 926 694 | This study |
| OEF553 | GTTCAATCGACGGATGTAA | 1 927 314 | This study |
| OEF554 | TAGCCTGCATTTGCTGAAG | 291 436 | This study |
| OEF555 | CTTTTGCGGTACCATTAACG | 291 919 | This study |
| OEF557 | GCAACAGATGCTAATGGAG | 2 737 353 | This study |
| OEF558 | GCCAATCCATTTAACGAAGC | 2 703 739 | This study |
| OEF559 | GTATCCTATCGATGGTGTG | 2 704 251 | This study |
| OEF560 | TCGAAGGTTCCTGATGAAC | 2 817 577 | This study |
| OEF561 | ACTGCTCTCACCAAATGTAG | 2829302 | This study |
| OEF564 | AGCCAATTGGTAACGTCCAC | 2 817 997 | This study |
| OEF565 | TTCAGCTCCTAATCTAGTAG | 2 818 606 | This study |
| OEF573 | ATGCAGACTACCAAGTCATG | 323 573 | This study |
| OEF574 | ATCATGTGCATAGCCAAAGC | 325 002 | This study |
| OEF575 | AGATTGTTGTGAAGCGAACG | 1 443 399 | This study |
| OEF576 | TGAGCAACTTAAAGGAGGTG | 1 441 968 | This study |
| OEF577 | TCAGCACGTTCATTTAATCC | 1 958 890 | This study |
| OEF578 | ATTCTTCGCAAATTTGACGG | 1 960 329 | This study |
| OEF579 | GATCAGAATGGGTAGCTAAG | 2 732 677 | This study |
| OEF580 | ATCGTCAGATGGTTTAGCAC | 2 734 199 | This study |
| OEF581 | ACATCCGTCATTGACTTACG | 2 825 960 | This study |
| OEF582 | CTATTAAGAGTGGAACGTGG | 2 827 442 | This study |
| OEF585 | GTACGGTTGGATTAACGAAC | 2 816 552 | This study |
| OEF591 | CGGAAGCAAGAGTTGAAAGC | 1 255 569 | This study |
| OEF592 | TTGCCAATCGGACCAAACG | 1 259 906 | This study |
| OEF618 | TAGCCATATGAGACGAAACG | 2 699 418 | This study |
| OEF619 | GTTAGATAGAGCCTAGAATC | 2 700 416 | This study |
| OEF620 | GATTCTAGGCTCTATCTAACTAAATTATTTAGTTTCACGGTG | 2 736 907 | This study |
| OEF621 | AACCATGCAATTAACTGCG | 2 737 969 | This study |
| OEF622 | AAACGATTGATAGTGAACCG | 2 699 195 | This study |
| OEF623 | TGGAGAAGTCACACCTAATC | 2 738 142 | This study |
| OEF624 | ACGATGTTACTCGCCTAAC | 2 700 282 | This study |
| OEF626 | AATAACGTACCCGTCTTTTC | 288 080 | This study |
| OEF627 | TGCCAAAACAGTTGGCGC | 289 226 | This study |
| OEF628 | GCGCCAACTGTTTTGGCACCTTGGGATCCAATGGGCGC | 326 495 | This study |
| OEF629 | TTGATTGATGCTGAAGGTAG | 327 598 | This study |
| OEF630 | CGTAAAATGAAAGGACGATG | 287 920 | This study |
| OEF631 | TGACAATCAACGTTACCAAC | 327 779 | This study |
| OEF634 | TATGTATAATCGAGGGTCAC | 1 921 627 | This study |
| OEF635 | CAAATATACGAAGAAAATTAAC | 1 922 596 | This study |
| OEF636 | GTTAATTTTCTTCGTATATTTGCCACTCCCCATCTGAAATTG | 1 962 541 | This study |
| OEF637 | ATTTGATGCGCCATACAACC | 1 963 549 | This study |
| OEF638 | TGGGAACAAATTAGCACCTC | 1 921 400 | This study |
| OEF639 | GTCCATACATTCTGGTTACC | 1 963 762 | This study |
| OEF640 | GAATATCCCTGCTATCACAC | 1 922 523 | This study |
| OEF641 | GGTTGTAATAGCTGTGATTCC | 2 815 695 | This study |
| OEF642 | CACGTTGTTTCATTAATAAAT | 2 816 749 | This study |
| OEF643 | ATTTATTAATGAAACAACGTGTTTAATCATATAATAAACCAA | 2 829 750 | This study |
| OEF644 | AGTGTCAATCATCCGGAACTG | 2 830 756 | This study |
| OEF645 | GAAATGCTGTATGTCAATGGC | 2 815 501 | This study |
| OEF646 | ATCTTCTTGCCACGATTATCC | 2 830 927 | This study |
| OEF653 | AAGTGCCAACAATGGATGC | 1 397 790 | This study |
| OEF656 | TCATCATTGTACTCCACTCC | 2 048 416 | This study |
| OEF657 | ATCAGTGAAATGGTTGTTCG | 2 829 802 | This study |

* Sequences added for fusion PCR are underlined.

^†^ Position of the primer 5’ end in the V583 genome.
