# Supplementary material for: Enterococcus faecalis Prophage Dynamics and Contributions to Pathogenic Traits
Source: PLoS Genet. 2013 Jun 6;9(6):e1003539. doi: 10.1371/journal.pgen.1003539 (PMC3675006; doi:10.1371/journal.pgen.1003539)
Supplement: Table S3 — V583 phages infection and immunity. (DOC) [file pgen.1003539.s007.doc]

**Table S3** V583 phages infection and immunity.

| **Indicator strain** | **Lysate** | |  | |  | |  | |  | |  | |  | |  | |  | |
| --- | --- | --- | --- | --- | --- | --- | --- | --- | --- | --- | --- | --- | --- | --- | --- | --- | --- | --- |
|  | **WT_37°C_** | **WT_42°C_** | | ***pp3ˉpp5ˉ*** | | ***pp1^+^*** | | ***pp3^+^ pp5^+^*** | | ***pp4^+^*** | | ***pp6^+^*** | | ***pp7^+^*** | | ***pp1^+^ pp7^+^*** | |  |
| *ppˉ* | + | + | | + | | + | | + | | - | | - | | - | | + | |  |
| *pp1ˉ* | - | - | | - | | - | | - | | - | | - | | - | | - | |  |
| *pp3ˉ* | + | + | | - | | - | | + | | - | | - | | - | | - | |  |
| *pp4ˉ* | - | - | | - | | - | | - | | - | | - | | - | | - | |  |
| *pp5ˉ* | + | + | | - | | - | | + | | - | | - | | - | | - | |  |
| *pp6ˉ* | - | - | | - | | - | | - | | - | | - | | - | | - | |  |
| *pp7ˉ* | + | + | | + | | - | | - | | - | | - | | - | | + | |  |
| *pp1ˉ pp7ˉ* | + | + | | + | | + | | - | | - | | - | | - | | + | |  |
| *pp3ˉ pp5ˉ* | + | + | | - | | - | | + | | - | | - | | - | | - | |  |
| *pp1^+^* | + | + | | + | | - | | + | | - | | - | | - | | + | |  |
| *pp3^+^ pp5^+^* | + | + | | + | | + | | - | | - | | - | | - | | + | |  |
| *pp4^+^* | + | + | | + | | + | | + | | - | | - | | - | | + | |  |
| *pp6^+^* | + | + | | + | | + | | + | | - | | - | | - | | + | |  |
| *pp7^+^* | + | + | | + | | - | | + | | - | | - | | - | | - | |  |

+ Plaque formation detection

- No plaque formation detection
